# Microbial Mat Compositional and Functional Sensitivity to Environmental Disturbance

**DOI:** 10.3389/fmicb.2016.01632

**Published:** 2016-10-17

**Authors:** Eva C. Preisner, Erin B. Fichot, Robert S. Norman

**Affiliations:** ^1^Department of Environmental Health Sciences, University of South Carolina, ColumbiaSC, USA

**Keywords:** microbial mat, rare biosphere, ecosystem, salinity, disturbance

## Abstract

The ability of ecosystems to adapt to environmental perturbations depends on the duration and intensity of change and the overall biological diversity of the system. While studies have indicated that rare microbial taxa may provide a biological reservoir that supports long-term ecosystem stability, how this dynamic population is influenced by environmental parameters remains unclear. In this study, a microbial mat ecosystem located on San Salvador Island, The Bahamas was used as a model to examine how environmental disturbance affects the protein synthesis potential (PSP) of rare and abundant archaeal and bacterial communities and how these changes impact potential biogeochemical processes. This ecosystem experienced a large shift in salinity (230 to 65 g kg^-1^) during 2011–2012 following the landfall of Hurricane Irene on San Salvador Island. High throughput sequencing and analysis of 16S rRNA and rRNA genes from samples before and after the pulse disturbance showed significant changes in the diversity and PSP of abundant and rare taxa, suggesting overall compositional and functional sensitivity to environmental change. In both archaeal and bacterial communities, while the majority of taxa showed low PSP across conditions, the overall community PSP increased post-disturbance, with significant shifts occurring among abundant and rare taxa across and within phyla. Broadly, following the post-disturbance reduction in salinity, taxa within Halobacteria decreased while those within Crenarchaeota, Thaumarchaeota, Thermoplasmata, Cyanobacteria, and Proteobacteria, increased in abundance and PSP. Quantitative PCR of genes and transcripts involved in nitrogen and sulfur cycling showed concomitant shifts in biogeochemical cycling potential. Post-disturbance conditions increased the expression of genes involved in N-fixation, nitrification, denitrification, and sulfate reduction. Together, our findings show complex community adaptation to environmental change and help elucidate factors connecting disturbance, biodiversity, and ecosystem function that may enhance ecosystem models.

## Introduction

Many ecosystems are in a continuous state of change due to diel, seasonal, and intermittent extreme weather-driven fluctuations in abiotic factors (e.g., nutrients, pH, light, temperature, and salinity). The ability of ecosystems to adapt to these changes depends on the duration and intensity of change and the biological diversity of the system (Sousa, 1984; Glasby and Underwood, 1996; Scheffer et al., 2001; Fraterrigo and Rusak, 2008). Significant biological diversity often exists within microbial communities controlling the biogeochemical processes that form the foundation of ecosystems (Falkowski et al., 2008); therefore, understanding the complex links between environmental change and microbial diversity is essential for assessing ecosystem stability (i.e., biogeochemical cycling). Studies examining these links within lake (Shade et al., 2011, 2012), marine sediment (Mohit et al., 2015), soil (Allison and Martiny, 2008), and microbial mat (Boujelben et al., 2012) ecosystems have shown that microbial communities are seasonally variable and often show long-term resilience to larger environmental disturbances. Further insight into the complex dynamics and ecological mechanisms maintaining ecosystems has revealed that while much of the microbial biomass is contained within a few dominant taxa, the greatest genetic diversity and metabolic potential is maintained within a vast number of low abundance or ‘rare’ taxa (Sogin et al., 2006). Studies examining the significance of these rare taxa have shown that while some may be active and contribute to important biogeochemical processes (Pester et al., 2010), others are less active and may be providing a genetic reservoir (i.e., seedbank) to maintain ecosystem diversity over time (Jones and Lennon, 2010; Lennon and Jones, 2011). For instance, resuscitation of rare taxa has been suggested to contribute to soil ecosystem functioning (Aanderud et al., 2015). However, the links between shifting environmental parameters and regulation of this dynamic population are unclear and an area of more recent research.

Complex semi-closed organo-sedimentary microbial mat ecosystems contain tightly coupled biogeochemical processes along a narrow vertical chemical gradient often occurring within the first 10 mm of microbial mats (Pinckney et al., 1995; Woebken et al., 2015), allowing them to serve as unique model systems to examine how environmental parameters influence the abundance and potential activity of rare taxa and biogeochemical cycling. While specific responses of the microbial mat rare biosphere to environmental change have yet to be explored, studies have revealed complex community dynamics with taxon-specific responses at different salinities. For instance, Cyanobacteria were shown to be tolerant to a range of salinities (Green et al., 2008), and while oxygenic and anoxygenic phototrophs tolerate a wide range of salinities up to 300 g kg^-1^ (Oren, 1999), in solar salterns, optimal growth of anoxygenic phototrophs has been detected at 100–120 g kg^-1^ and sulfate-reducing bacteria (SRB) are poorly adapted to salinities over 200 g kg^-1^ (Sorensen et al., 2004). With processes within these ecosystems tightly coupled, salinity-driven shifts in community structure will alter biogeochemical cycling (Ley et al., 2006). For instance, increased salinity decreases nitrogen fixation by Cyanobacteria but increases fixation by Deltaproteobacteria (Severin et al., 2012). While studies have shown that microbial mat communities contain similar phyla with slight biogeographical and salinity-driven compositional differences (Ley et al., 2006; Allen et al., 2009; Dillon et al., 2013; López-López et al., 2013; Schneider et al., 2013), there is little knowledge regarding the complex compositional and functional dynamics occurring within abundant and rare taxa following an environmental disturbance and how these community changes may alter biogeochemical cycling.

This study provides knowledge on microbial mat compositional and functional sensitivity following a pulse disturbance resulting in a large salinity shift. While studies have shown that salinity is one of the most important parameters influencing global patterns of Archaea and Bacteria distribution (Lozupone and Knight, 2007; Auguet et al., 2010; Canfora et al., 2014), these studies often compare communities isolated and adapted to a range of stable salinities. The mat ecosystem investigated here experiences routine seasonal shifts in salinity and extreme disturbances following hurricanes and tropical storms, providing an opportunity to examine the response of a semi-closed microbial community to environmental disturbance. Sequencing of archaeal and bacterial 16S rRNA and rRNA genes showed significant shifts in the day/night protein synthesis potential (PSP) of abundant and rare taxa following the reduction in salinity from 230 to 65 g kg^-1^ that occurred between 2011 and 2012 following the landfall of Hurricane Irene. Quantitative PCR of genes and transcripts involved in nitrogen and sulfur cycling show concomitant shifts in gene expression indicating a possible change in biogeochemical cycling potential. Together, these data show the compositional and functional sensitivity of a microbial mat ecosystem to environmental change but also suggest that rare taxa may provide a reservoir of genetic diversity that enhances ecosystem stability following seasonal and extreme environmental disturbances.

## Materials and Methods

### Sample Collection and Processing

The mat ecosystem examined in this study is located on San Salvador Island, Bahamas (Supplemental Figure S1). The mat forms well-defined layers and experiences wide ranges in salinity (35 to >305 g kg^-1^), intense irradiance (>2200 μmol m^-2^ s^-1^), and high temperatures (>40°C; Pinckney et al., 1995; Pinckney and Paerl, 1997; Paerl et al., 2003, personal observation).

Mat cores were obtained from similar locations during August 1–2, 2011 (230 g kg^-1^salinity) and 2012 (65 g kg^-1^ salinity). To focus on the most diverse component of the consolidated microbial mat, samples (0.9–1.5 g) of the top 7 mm of the mat were taken during daytime (10am/5pm) and nighttime (10pm/5am) using a 7-mm Harris Uni-Core^TM^ device (Ted Pella, Inc, Redding, CA, USA). Five replicate cores were pooled immediately (<1 min) into a 3-ml tube containing 2 ml RNAprotect Bacteria Reagent (Qiagen, Valencia, CA, USA). Triplicate tubes were obtained for each time point, resulting in 6 daytime and 6 nighttime replicates. Samples were homogenized with sterile glass rods and stored at 4°C until further processing. Environmental parameters were measured with an YSI 30 Salinity Meter, a YSI 55 Dissolved Oxygen Meter (YSI, Yellow Springs, OH, USA), a LI-COR LI-250A Light Meter (LI-COR, Lincoln, NE, USA), and a Mettler Toledo SevenGo Portable pH Meter (Mettler-Toledo, Columbus, OH, USA).

### Nucleic Acid Extraction and cDNA Synthesis

Mat samples in RNAprotect Bacteria reagent were centrifuged (8,000 × *g* for 5 minutes) and RNAprotect discarded. The pellet was resuspended in 7.5 ml RLT Plus buffer (Qiagen) containing 1% 2-mercaptoethanol. Samples were incubated at room temperature for 10 min followed by five freeze/thaw cycles consisting of freezing in liquid nitrogen and thawing at 55°C. Next, silicon carbide beads (DNase- and RNase-free mixture of 0.1 and 1 mm beads) were added and samples vortexed for 10 min and processed using the Allprep DNA/RNA Miniprep Kit (Qiagen) for simultaneous DNA/RNA extraction from each sample. Total RNA and DNA were quantified using a Qubit 2.0 fluorometer (Life technologies, Grand Island, NY, USA). Turbo DNA-free kit (Ambion, ThermoFisher Scientific) was used to remove DNA from total RNA and DNA removal verified through PCR. RNA (100 ng) was reverse transcribed to cDNA using SuperScript III first-strand synthesis (Life Technologies) with random hexamers.

### 16S rRNA/rRNA Gene Amplification and Sequencing

The abundance and PSP of archaeal and bacterial communities under different salinities were assessed by sequencing and analysis of 16S rRNA and rRNA genes. For clarity, when comparing 16S rRNA and rRNA gene-based data, these data will be referred to as 16S rRNA and rDNA, respectively. For Bacteria, the V1–V3 hypervariable region was amplified using a combination (1:1 molar ratios) of the 27F forward primer (AGA GTT TGA TCC TGG CTC AG; Edwards et al., 1989) and the 27Fd forward primer (AGA GTT TGA TYM TGG CTC AG; Nercessian et al., 2005) and the U529 universal reverse primer (ACC GCG GCK GCT GRC; Marshall et al., 2012). For Archaea, the V2–V3 hypervariable region was amplified using the forward primer 109F (ACK GCT CAG TAA CAC GT; Whitehead, 1999) and the U529 reverse primer. Twelve multiplex identifier (MID) tags were added to reverse primers for sample multiplexing (Supplemental Table S1).

Triplicate 25 μl PCR reactions contained 0.625 units of GoTaq^®^ Hot Start Polymerase (Promega Corp., Madison, WI, USA), 1.5 mM MgCl_2_, 0.2 mM nucleotide mix, 0.3 μM each primer, and 10 ng template DNA or cDNA. PCR conditions consisted of: initial denaturation: 95°C for 5:00 min; 94°C for 0:45 min, annealing at 62°C for 0:45 min using -0.5°C per cycle, and elongation at 72°C for 0:45 min, for 10 touchdown cycles; (94°C for 0:45 min, 62°C -0.5°C per cycle for 0:45 min, and 72°C for 0:45 min); 25 (for Archaea) or 15 (for Bacteria) additional cycles (94°C for 0:45 min, 57°C for 0:45 min, and 72°C for 0:45 min); final elongation at 72°C for 10:00 min. Amplicons were purified using the QIAquick PCR purification kit (Qiagen) and quantified with a Qubit fluorometer.

For each sample, bacterial and archaeal amplicons with similar MIDs were combined (2:1) and uniquely labeled triplicates for each time point combined (1:1) before Illumina library preparation. Four libraries (2011 rDNA, 2012 rDNA, 2011 rRNA, and 2012 rRNA) were prepared using the NEBNext^®^ DNA Library Prep Master Mix (New England Biolabs, Ipswich, MA, USA). The libraries were combined (1:1) and sequenced on an Illumina MiSeq using the MiSeq Reagent Kit v3 (Illumina, Inc., San Diego, CA, USA). FASTQ formatted paired end reads are located in the GenBank sequence read archive under SRP070186.

### Sequence Processing

Sequences were analyzed using mothur [v.1.33.0; (Schloss et al., 2009)] following a modified version of the MiSeq SOP (Kozich et al., 2013). After paired-end reads from all libraries were assembled, sequences not matching quality criteria (maximum ambiguities = 0, ≤8 homopolymers, ambiguous length ≥300 or <650 bp) were culled using the screen.seqs command. Sequences were demultiplexed, MIDs trimmed, and separated into Archaea and Bacteria based on classification (RDPclassifier.trainset9). Non-chimeric sequences were dereplicated and aligned using Silva Archaea and Bacteria databases trimmed to the V1–V3 region. Sequences were assigned to operational taxonomic units (OTUs) with a sequence similarity threshold of 97% identity and classified. For increased stringency, OTUs represented by <20 sequences, across groups (i.e., day and night: 2011 rRNA, 2011 rDNA, 2012 rRNA, and 2012 rDNA) were culled before further analysis. Sequences were rarefied (Bacteria = 27,663 per MID, Archaea = 24,390 per MID) and beta diversity estimated using 𝜃_Y C_ at a 0.03 distance threshold and visualized using non-metric multidimensional scaling (NMDS). The three dimensions of the NMDS data were plotted in SigmaPlot. Analysis of molecular variance (AMOVA) was performed to test the significance of variation among conditions.

### PSP of Rare/Abundant Taxa

Linear discriminant analysis effect size (LEfSe, LDA > 2, and *n* = 6 per condition) identified statistically significant OTUs among replicates within pre- and post-disturbance salinity conditions (Schloss et al., 2009; Segata et al., 2011). From initial data, 96% of archaeal and 98% of bacterial OTUs were identified as significant among conditions and used for subsequent analysis. The relative abundance (%) of OTUs within conditions was calculated and classified as “rare” when abundance was <1%, and “abundant” when >1%. Archaeal and bacterial OTU frequencies were square root transformed using SPSS Statistics 22 (IBM Corp., Armonk, NY, USA) to better visualize data distribution. Nonparametric correlations (Kendall’s τ and Spearman’s ρ) were calculated using transformed data of relative 16S rRNA and rDNA frequencies from 2011 and 2012 samples using SPSS and frequencies plotted with SigmaPlot. The PSP of OTUs was determined by calculating 16S rRNA:rDNA ratios. While this approach may not distinguish between extremely slow-growing and dormant taxa, comparison of the ratio for individual OTUs across salinities is used throughout this study to indicate increased or decreased PSP for given OTUs. Mean (*n* = 6) PSP for Archaea classes and Bacteria phyla were calculated and reported with its standard error. One-way analysis of variance (ANOVA) was used to determine if there was a significant (*p* = 0.05) PSP difference between mean PSP values for each condition (Day11, Night11, Day12, and Night12) and a student’s *t*-test for observing significant changes between pre- and post-disturbed conditions using SPSS statistics. Heatmaps were used to visualize mean (*n* = 6) rRNA:rDNA ratios for OTUs across conditions and constructed within RStudio with the heatmap2 command in gplot (Warnes et al., 2012).

### Quantitative PCR

Abundance of genes and transcripts involved in nitrogen and sulfur cycling as well as overall Archaea and Bacteria abundance was measured by quantitative PCR (qPCR). To generate qPCR standard curves, each target gene was initially amplified from either combined 2011/2012 DNA or positive isolate controls. Amplicons were size verified, ligated into pCR2.1-TOPO cloning vectors (Invitrogen), and transformed into One Shot *E. coli* DH5α-T1-resistant competent cells according to the manufacturer’s instructions. Plasmids from selected clones were extracted using the QIAprep spin mini kit (Qiagen) and target validated by DNA sequencing (Macrogen USA, MA, USA) of the insert and BLASTX comparison against the NCBI NR database. After linearization of plasmids with HindIII endonuclease (New England BioLabs Inc., Ipswich, MA, USA), plasmid concentrations were measured fluorometrically with a Qubit fluorometer (Life technologies) and serially diluted resulting in standards ranging from 10^3^ to 10^9^ copies μl^-1^. For each target, triplicate qPCR reactions were performed for each standard and biological triplicate in an ABI 7900HT Fast Real-Time PCR system (Applied Biosystems, Carlsbad, CA, USA) with a final volume of 25 μl, including 12.5 μl PerfeCta SYBR Green SuperMix, Rox (Quanta Biosciences, Gaithersburg, MD, USA), 300 nM respective primers (Supplemental Table S2), and 10 ng DNA/RNA (cDNA). Template DNA/RNA consisted of pooled (equal molar) day and night nucleic acids to examine overall day/night biogeochemical cycling and domain distribution during sampling years. The efficiency of each qPCR was calculated and ranged between 90 and 100%. Data were analyzed with SPSS using an independent sample *t*-test (two-tailed, *p* < 0.05) to detect differences in RNA:DNA ratios between 2011 and 2012, as well as multiple comparison analysis (ANOVA) of 16S rRNA genes to detect differences in Archaea and Bacteria abundances.

## Results

### Salt Pond Environmental Conditions

Measurements of the environmental parameters at Salt Pond during August 2011 showed the following day/night pre-disturbance conditions: salinity 230 g kg^-1^, water temperature 40/25°C, dissolved oxygen 2.45/1.45 mg/L, and pH 7.6/6.8 (Supplemental Figure S1). The landfall of Hurricane Irene provided a disturbance to the ecosystem, resulting in the following August 2012 post-disturbance day/night conditions: salinity 65 g kg^-1^, water temperature 35/30°C, dissolved oxygen 3.08/2.30 mg/L, and pH 7.5/7.3. The results outlined below describe the Salt Pond microbial mat community response to this ecosystem disturbance.

### Post-disturbance Shifts in Community Diversity

Following the reduction in salinity, qPCR of 16S rRNA genes from replicate day/night samples indicated significant (*p* < 0.05) changes in overall domain prevalence with Archaea decreasing from 57 to 33% and Bacteria increasing from 43 to 67%. To further explore differences in community composition (β-diversity) between salinity conditions, 𝜃_Y C_ distances were visualized on NMDS plots (Supplemental Figure S2). Distribution of 16S rDNA and rRNA-based distances showed uniform clustering of Archaea and Bacteria day and night samples within respective years, suggesting minimal diel influence on community composition. However, spatial separation was observed between years as 2011 and 2012 samples formed distinct clusters, indicating an overall shift in the archaeal and bacterial communities with decreased salinity. Analysis of molecular variance (AMOVA) further supported the significant difference in community composition between years (*p* < 0.0001). Further comparison between the distribution of rDNA and rRNA-based distances showed uniform clustering for archaeal samples under both salinity conditions, indicating similar rDNA and rRNA-based community structure. However, within the bacterial community, rDNA- and rRNA-based samples formed unique clusters under the pre-disturbance high saline conditions of 2011 as compared to more even distribution observed after the post-disturbance reduction in salinity.

### Post-disturbance Shifts in Archaeal Abundance

**FIGURE 1:**
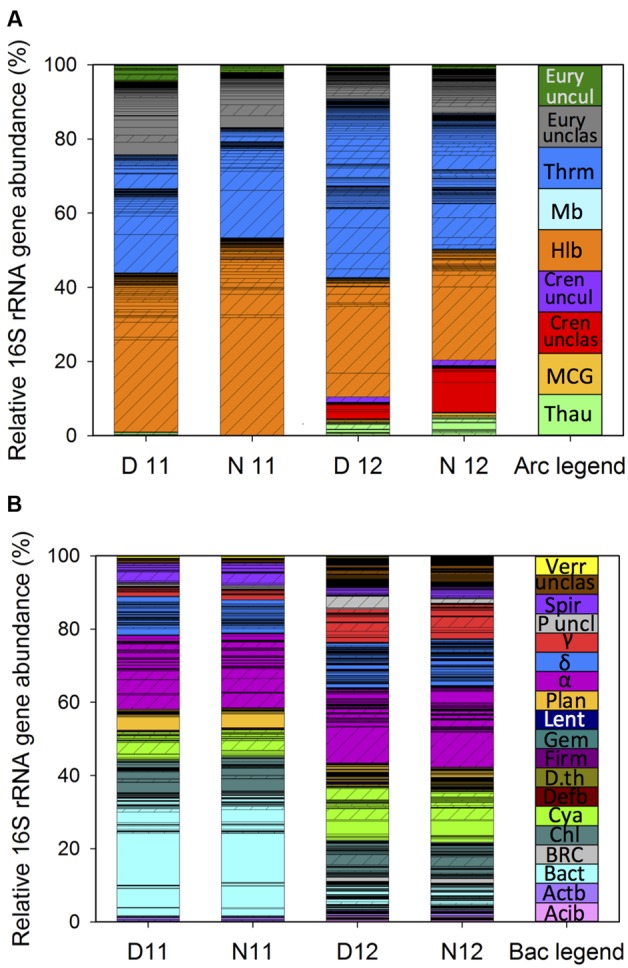
**Stacked bar charts of microbial mat community profiles for Archaea **(A)** and Bacteria **(B)** based on relative 16S rRNA gene abundance of operational taxonomic units (OTUs) sorted by phylogenetic classification during day and night under high (2011) and lower salinity (2012).** Bars represent the average (*n* = 6) relative 16S rRNA gene abundance of each OTU under the following conditions: D11, day 2011; N11, night 2011; D12, day 2012; N12, night 2012. Standard deviations for OTUs are located in the Supplemental Table S4. Cross hatched bars represent OTUs present under both salinities. Phylogenetic classification was made with Silva archaea or bacteria reference files using the 16S rRNA gene MiSeq sequencing data. **(A)**: Thau, Thaumarchaeota; MCG, Miscellaneous Crenarchaeotic Group; Cren unclas, Crenarchaeota unclassified; Halo, Halobacteria; Mb, Methanobacteria; Thrm, Thermoplasmata; Eury unclas, Euryarchaeota unclassified; Eury uncul, Euryarchaeota uncultured. **(B)**: Acib, Acidobacteria; Actb, Actinobacteria; Bact, Bacteroidetes; BRC1, Bacteria Candidate division BRC1; Chl, Chloroflexi; Cya, Cyanobacteria; Defb, Deferribacteria; D.th, Deinococcus Thermu*s*, Firm, Firmicute*s*; Gem, Gemmatimonadetes, Lent, Lentisphaerae; Plan, Planctomycetale*s*; α, Alphaproteobacteria, δ, Deltaproteobacteria; γ, Gammaproteobacteria; P uncl, Proteobacteria unclassified; Spiro., Spirochaetales; unclas, Bacteria unclassified; Verr, Verrucomicrobia.

To better understand how the archaeal community shifted following the reduction in salinity, the relative abundance of archaeal 16S rRNA genes was compared across years (**Figure 1A**). The relative abundance for each OTU represents the average among six biological replicates and only OTUs that were determined to be significant through LEfSe analysis are displayed. The standard deviation for each OTU is listed in the Supplemental Table S4. Furthermore, due to similarities in day/night abundances, average abundances are discussed for comparison between years. During both pre- and post-disturbance years, approximately 4% of archaeal OTUs had relative sequence abundances >1% and classified as ‘abundant’ while the majority were <1% and classified as ‘conditionally rare’. Although rare OTUs comprised a majority of the community richness, in total they represented only 33 and 39% of overall archaeal 16S rRNA gene abundance in 2011 and 2012, respectively. Following the reduction in salinity, OTU abundance shifted with 4 OTUs remaining abundant across salinities, while 3 shifted from abundant to rare and 11 from rare to abundant. Additionally, 214 OTUs (46%) were detected only in the post-disturbance samples, suggesting greater environmental specialization by taxa than that suggested by the lower-resolution NMDS plots.

Classification of OTUs showed that under the 2011 high salt conditions, the single most abundant class was Halobacteria, representing approximately 45 ± 0.12% of total archaeal 16S rRNA gene abundance (**Figure 1A**, D/N11). While most abundant, the evenness of the Halobacteria community was skewed toward a single OTU belonging to the family, Halobacteriaceae. While this OTU remained the most abundant in post-disturbance samples, an overall onefold reduction in Halobacteria OTU richness was observed, resulting in a 0.7-fold decrease in Halobacteria relative abundance (**Figure 1A**; D/N12). The next most abundant classes identified under high salinity were unclassified Euryarchaeota and Thermoplasmata, which showed the highest OTU richness across years but only represented approximately 16.55 ± 0.03 and 29.44 ± 0.10% of overall archaeal 16S rRNA gene abundance, respectively (**Figure 1A**; D/N11). Following salinity reduction, previously abundant Thermoplasmata OTUs decreased while previously rare or not detected OTUs expanded, resulting in a 0.7-fold increase in richness and a 0.5-fold increase in relative abundance (**Figure 1A**; D/N12). Within the archaeal community, the greatest shift in relative abundance was observed for Crenarchaeota, with unclassified and uncultured Crenarchaeota increasing approximately sixfold in post-disturbance samples (**Figure 1A**). While few archaeal OTUs across salinities classified within the Thaumarchaeota phylum, an increase in richness and abundance was observed with the reduction in salinity.

### Post-disturbance Shifts in Bacterial Abundance

To examine post-disturbance shifts in the bacterial community, the relative abundance of bacterial 16S rRNA genes was also compared across years (**Figure 1B**). As above, the relative abundance for each OTU represents the average among replicates and only significant OTUs are displayed with standard deviations listed in the Supplemental Table S4. Similar to the archaeal community, during both years, approximately 2% of bacterial OTUs were classified as ‘abundant’ while the remaining were classified as ‘conditionally rare’. While rare OTUs comprised a majority of the community richness, in total they represented 46 and 65% of relative bacterial 16S rRNA gene abundance in 2011 and 2012, respectively. Following the reduction in salinity, OTU abundance shifted with 2 OTUs remaining abundant across both salinities, while 3 shifted from abundant to rare and 6 from rare to abundant. In addition, 625 OTUs (61%) were only detected in the post-disturbance samples, suggesting environmental specialization by many bacterial taxa.

Classification of bacterial OTUs indicated that Bacteroidetes was the most abundant pre-disturbance phylum with three OTUs classified as Sphingobacteria having the greatest abundance (**Figure 1B**; D/N11). Under post-disturbance conditions, while Bacteroidetes richness remained the same, relative abundance decreased by twofold (**Figure 1B**, D/N12). For the following phyla, Actinobacteria, Chloroflexi, Planctomycetes, and Verrucomicrobia, equal abundance was observed across years, however, some OTUs were only present under one salinity condition. For instance, within the phylum Planctomycetes, one OTU classified as Phycisphaerae was abundant under high salinity and decreased significantly following the shift in salinity. In addition, while all Planctomycetes OTUs belonged to the rare population in 2012, more than half were detected only at lower salinity, resulting in overall increased phylum richness. Cyanobacteria comprised approximately 8% (±1) of the pre-disturbance bacterial 16S rRNA gene abundance and increased by 0.8-fold concomitant with salinity reduction (**Figure 1B**). Within the Cyanobacteria, while most OTUs were classified as conditionally rare, one was abundant under both conditions while two changed from not detected or rare to abundant with salinity reduction. The greatest bacterial community richness across both salinities was contained within the Proteobacteria phylum. Within this phylum, Alphaproteobacteria was the most abundant class across years, followed by Delta-, Gamma-, and unclassified-Proteobacteria. Furthermore, among the Alphaproteobacteria, four OTUs belonging to the orders Rhizobiales, Rhodobacterales, and unclassified Alphaproteobacteria were the most abundant under high salinity. Following the reduction in salinity, Rhodobacterales OTUs decreased by 0.8-fold, while a single abundant Rhizobiales OTU further increased by onefold, shifting the order to become the most abundant Alphaproteobacteria at lower salinities. While Deltaproteobacteria had the greatest richness among the Proteobacteria, with OTUs spanning nine orders, the Desulfobacterales and unclassified Deltaproteobacteria contained the most OTUs and had the highest abundance within the class. While no change in overall abundance was observed across salinities, OTU distribution among Deltaproteobacteria changed significantly, with half of the OTUs detected only under one condition (**Figure 1B**, non-cross hatched OTUs). The largest shift in proteobacterial abundance across salinities was observed within the Gammaproteobacteria, which increased approximately threefold following the decrease in salinity. This shift in abundance was correlated with an increase in three OTUs classified as Chromatiales and Sedimenticola.

### Post-disturbance Shifts in Archaeal 16S rRNA:rDNA Ratios

**FIGURE 2:**
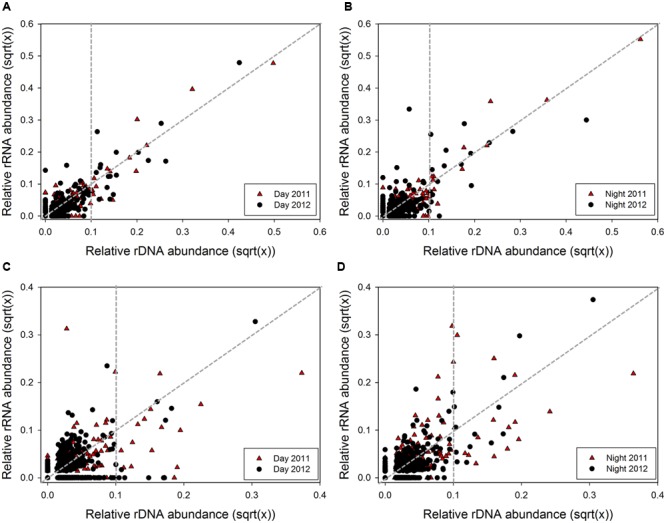
**Relationship between rRNA and rDNA abundance for each OTU defined by 16S rRNA and 16S rRNA gene MiSeq sequencing dataset for subsampled communities comparing 2011 (black) and 2012 (red).** Archaeal **(A)** day (*n*_day2011_= 277, *n*_day2012_= 330) and **(B)** night (*n*_night2011_= 249, *n*_night2012_= 367); and bacterial **(C)** day (*n*_day2011_= 372, *n*_day2012_= 697), and **(D)** night (*n*_night2011_= 347, *n*_night2012_= 621). Data points in graph are square root transformed paired relative rRNA and rDNA abundances for each OTU. Vertical gray dashed line differentiates between rare and abundant OTUs (rare < 0.1%, abundant > 0.1% rDNA abundance). Data points above and below the 1:1 line represent OTUs with higher and lower PSP, respectively.

Correlation analysis was used to test the association between 16S rRNA and rDNA abundance during day and night across conditions (**Figure 2**). A positive correlation between rRNA and rDNA abundance was observed for OTUs during daytime across both years (**Figure 2A**; Kendall’s nonparametric τ_day2011_ = 0.59, *p* < 0.0001, *n* = 275; τ_day2012_ = 0.59, *p* < 0.0001, *n* = 330). Similarly, while a positive correlation was also observed during nighttime, a reduction in the τ coefficient during 2012 suggested a weaker association under the lower salinity nighttime samples (**Figure 2B**; Kendall’s nonparametric τ_night2011_ = 0.55, *p* < 0.0001, *n* = 249; τ_night2012_= 0.38, *p* < 0.0001, and *n* = 367). Further analysis showed that most archaeal OTUs had rRNA:rDNA ratios below the 1:1 correlation line under all conditions (**Figures 2A,B**). However, following the salinity reduction in 2012, the total number of abundant and rare OTUs with ratios above the 1:1 line increased by 0.2-fold, indicating an overall increase in archaeal PSP.

**FIGURE 3:**
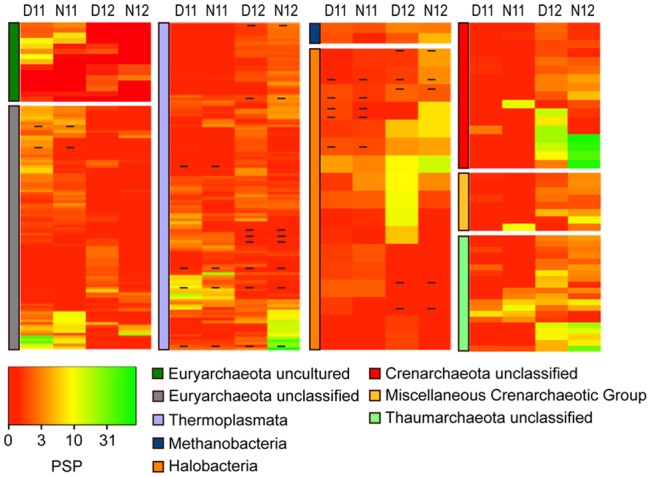
**Heatmap analysis of OTU PSP for each archaeal class for day and night samples across years (D11, Day 2011; N11, Night 2011, D12, Day 2012; N12, Night 2012).** The heatmap color represents the extent of protein synthesis potential (PSP) as measured by rRNA: rDNA ratio for each OTU. Square colors shifted toward brighter green indicate higher PSP of that OTU. Dash within squares indicates abundant OTUs. Standard deviations are located in Supplemental Table S3.

To further explore the trends observed in the correlation analysis, archaeal OTUs were classified and 16S rRNA:rDNA ratios compared during day/night across salinity conditions (**Figure 3**). Overall, dynamic shifts in ratios for abundant and rare OTUs within and across classes indicated complex archaeal community environmental adaptation. During the pre-disturbance conditions of 2011, the highest mean rRNA:rDNA ratios were observed within unclassified and uncultured Euryarchaeota, followed by Thermoplasmata, Methanomicrobia, and Thaumarchaeota, (**Figure 3**, D/N11; Supplemental Table S3). Following salinity reduction, a significant (*p* < 0.01) shift in mean rRNA:rDNA ratios was observed concomitant with changes in abundance (**Figure 1A**), which resulted in more even distribution of PSP across classes (**Figure 3**, D12 and N12). For instance, a decrease in mean ratios was observed within unclassified and uncultured Euryarchaeota while ratios increased for unclassified Crenarchaeota, Thermoplasmata, Thaumarchaeota, and miscellaneous crenarchaeotic group. These broad shifts resulted in a significant increase in mean archaeal PSP (Supplemental Table S3).

### Post-disturbance Shifts in Bacterial 16S rRNA:rDNA Ratios

As opposed to archaeal OTUs, no significant association between 16S rRNA and rDNA abundance was observed for bacterial OTUs during daytime across both salinity conditions (**Figure 2C**; Kendall’s nonparametric τ_day2011_ = 0.05, *p* = 0.15, and *n* = 434; τ_day2012_= 0.06, *p* = 0.05, and *n* = 697). However, a weak positive correlation was observed for nighttime samples across years (**Figure 2D**; Kendall’s nonparametric τ_night2011_= 0.35, *p* < 0.0001, and *n* = 347; τ_night2012_= 0.26, *p* < 0.0001, and *n* = 621). Similar to Archaea, most bacterial OTUs had rRNA:rDNA ratios below the 1:1 correlation line under all conditions (**Figures 2C,D**). However, following the reduction in salinity, daytime rRNA:rDNA profiles shifted, with the number of rare OTUs above the 1:1 line increased by 1.3-fold, indicating greater PSP within this bacterial population. A similar trend was observed in nighttime samples with a 0.9- and 0.3-fold increase in the number of abundant and rare OTUs with higher rRNA:rDNA ratios.

**FIGURE 4:**
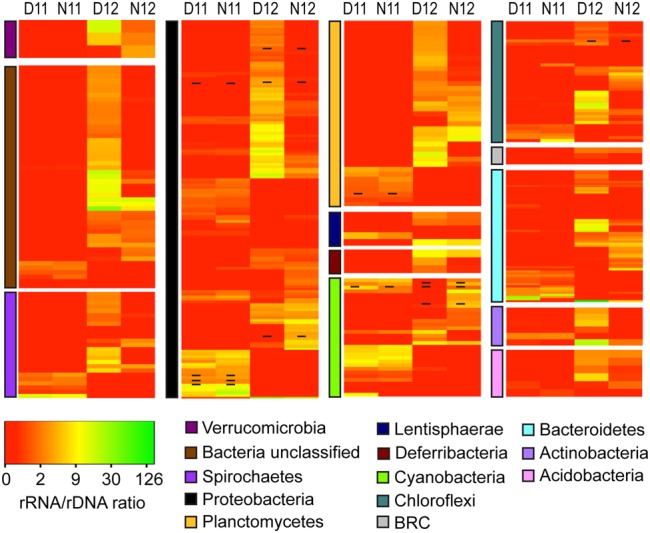
**Heatmap analysis of OTU PSP for each bacterial phylum for day and night samples across salinities (D11, Day 2011; N11, Night 2011; D12, Day 2012; N12 Night 2012).** The heatmap color represents the extent of PSP as measured by rRNA:rDNA ratio within each OTU. Square colors shifted toward brighter green indicate higher PSP of that OTU. Dash within squares indicates abundant OTUs. Standard deviations are located in Supplemental Table S3.

Classification and comparison of rRNA:rDNA ratios for bacterial OTUs across salinity and diel conditions indicated unique PSP profiles across and within phyla (**Figure 4**). Under the high salinity pre-disturbance conditions of 2011, rRNA:rDNA ratios were low for most OTUs with the highest ratios observed for OTUs within the Proteobacteria, Cyanobacteria, and Bacteroidetes phyla (**Figure 4**, D/N11; Supplemental Table S3). Following the reduction in salinity, increased rRNA:rDNA ratios were observed for rare OTUs among all phyla, indicating higher PSP across most phyla and an overall significant (*p* < 0.01) increase in bacterial PSP as determined by the mean day/night ratios for both salinity conditions (**Figure 4**, D/N12; Supplemental Table S3). Examination of diel trends across conditions showed that most phyla didn’t show significant differences in average day/night rRNA:rDNA profiles under the higher salinity conditions of 2011. However, under the lower salinity conditions of 2012, OTUs within most phyla showed decreased rRNA:rDNA ratios at night, resulting in a significant decrease in community mean PSP (*p* < 0.01) as compared to daytime (Supplemental Table S3).

**FIGURE 5:**
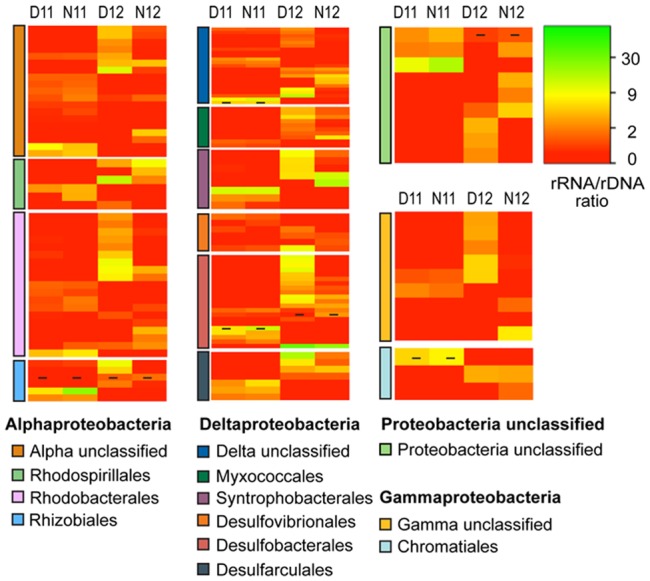
**Heatmap analysis of OTU PSP for each proteobacterial order for day and night samples across salinities (D11, Day 2011; N11, Night 2011; D12, Day 2012; N12 Night 2012).** The heatmap color represents the extent of PSP as measured by rRNA: rDNA ratio within each OTU. Square colors shifted toward brighter green indicate higher PSP of that OTU. Dash within squares indicates abundant OTUs. Standard deviations are located in Supplemental Table S3.

The Proteobacteria phyla was further resolved to better understand how the salinity shift affected rRNA:rDNA profiles of this metabolically diverse group (**Figure 5**). Following salinity reduction, increased rRNA:rDNA ratios were observed for OTUs within and across orders, resulting in a significant increase in mean daytime proteobacterial PSP. The Alphaproteobacteria was most abundant proteobacterial class across conditions (**Figure 1B**) and showed significant increase in mean PSP under lower salinity (**Figure 5**; Supplemental Table S3, Alphaproteobacteria). Among the Alphaproteobacteria, increased rRNA:rDNA ratios were observed for OTUs classified within the nitrogen fixing Rhizobiales and the purple non-sulfur bacteria, Rhodobacterales and Rhodospirillales. The Deltaproteobacteria had the highest OTU richness among proteobacterial classes (**Figure 1B**) and showed the greatest mean PSP in daytime 2012, with OTUs among all orders increasing rRNA:rDNA ratios following salinity reduction (**Figure 5**; Supplemental Table S3, Deltaproteobacteria). The Gammaproteobacteria had the greatest increase in abundance following salinity reduction due to the expansion of three OTUs contained within the Chromatiales and Sedimenticola orders (**Figure 1B**) which showed concomitant increases in rRNA:rDNA ratios (**Figure 5**, Gammaproteobacteria).

### Biogeochemical Cycling

#### Nitrogen Fixation

**FIGURE 6:**
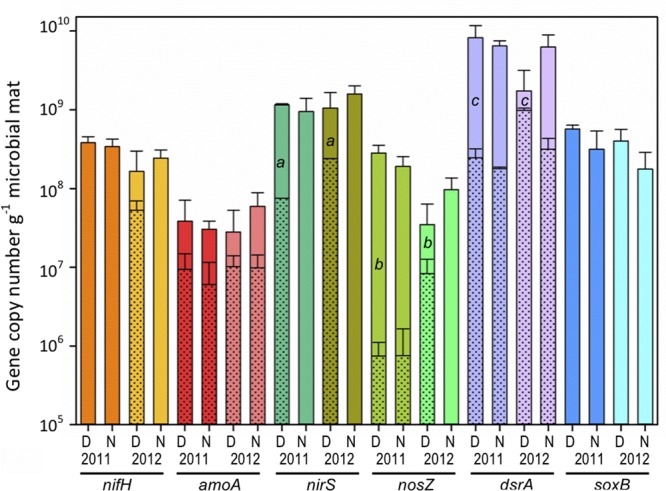
**Microbial mat community potential for biogeochemical cycling as determined by qPCR amplification of genes (DNA) and transcripts (cDNA) involved in nitrogen and sulfur cycling for day (D) and night (N) samples during 2011 (11) and 2012 (12) (*n* = 6).** Nitrogen cycling genes/transcripts analyzed: nitrogen fixation (*nif*H), archaeal nitrification (*amo*A), and denitrification (*nos*Z, and *nir*S). Sulfur cycling genes/transcripts analyzed: sulfate reduction (*dsr*A) and sulfide oxidation (*sox*B). Shaded areas within solid bars represent transcript abundance (no shade indicates that transcripts were not detected). Different lower case letters indicate significantly different cDNA/DNA ratios across years (independent *t*-test, *p* < 0.05).

The dinitrogenase reductase gene (*nifH*) abundance was measured to examine community nitrogen fixation potential (**Figure 6**). Across both pre- and post-disturbance conditions, no significant difference (*p* > 0.05) was observed in *nifH* DNA gene copy numbers per gram of microbial mat during day [2011: (3.8 ± 0.7) × 10^8^; 2012: (1.6 ± 1.4) × 10^8^] or night [2011: (3.4 ± 0.8) × 10^8^; 2012: (2.4 ± 0.7) × 10^8^], suggesting overall community genetic potential for nitrogen fixation remains similar across salinities (**Figure 6**, *nifH* D/N11 vs. D/N12). However, using the RNA/DNA ratio as a measure of potential *nifH* gene expression, an increase in daytime expression from below detection to 33% was observed following the reduction in salinity (**Figure 6**, *nifH* D11 vs D12 shaded bar). Nighttime *nifH* expression was below detection under both salinities.

#### Nitrification

Bacterial and archaeal ammonia monooxygenase genes (*amoA*) were analyzed to investigate the potential contribution of nitrification among ammonia-oxidizing Archaea (AOA) relative to ammonia-oxidizing Bacteria (AOB). Attempts to amplify bacterial *amoA* genes from pre- and post-disturbance conditions were unsuccessful and typical AOB taxa were not identified in Proteobacteria 16S rRNA or rRNA genes. However, archaeal *amoA* genes were identified in DNA at similar copy numbers with no significant difference (*p* > 0.05) across years [2011: day (3.8 ± 3.2) × 10^7^; night (3.0 ± 0.9) × 10^7^] and [2012: day (2.7 ± 2.5) × 10^7^); night (5.9 ± 6.7) × 10^7^] (**Figure 6**, *amoA* D/N11 vs. D/N12). Analysis of *amoA* gene expression during day and night showed average RNA/DNA ratios of 35% and 13% under high salinity conditions and increased to 63% and 20% following the 2012 post-disturbance salinity reduction.

#### Denitrification

The denitrification potential of the community was estimated through analysis of the *nirK*/*nirS* (nitrite reductase) and *nosZ* (nitrous oxide reductase) genes. Amplification of nitrite reductase genes from the Salt Pond microbial mat under both salinity conditions only identified *nirS* genes. Similar (*p* > 0.05) gene abundance for *nirS* and *nosZ* were observed during day [2011: *nirS* (1.2 ± 0.03) × 10^9^, *nosZ* (2.8 ± 0.7) × 10^8^; and 2012: *nirS* (1.1 ± 0.6) × 10^9^, and *nosZ* (3.5 ± 2.8) × 10^7^) and night (2011: *nirS* (9.5 ± 2.8) × 10^8^, *nosZ* (1.9 ± 0.6) × 10^8^; and 2012: *nirS* (1.6 ± 0.2) × 10^9^, and *nosZ* (9.7 ± 3.4) × 10^7^) under both salinity conditions (**Figure 6**). However, comparison of *nirS* to *nosZ* across both years indicated that the abundance of *nirS* was greater than *nosZ*. In addition, while *nirS* and *nosZ* gene abundances remained similar across years, significant increases (*p* < 0.001) in daytime RNA/DNA ratios for both genes were observed during 2012 (**Figure 6**, *nirS, nosZ*: D11 and D12). When examining potential differences in diel expression, *nirS* transcripts were below detection at night in both years while *nosZ* showed minimal (2.2%) nighttime expression during 2011 and below detection in 2012. Anaerobic ammonia oxidation (anammox) was also investigated and while taxa affiliated with the Plantomycetes phylum were identified, attempts to amplify the hydrazine oxidoreductase (hzo) genes were unsuccessful.

#### Sulfate Reduction

In this study, the dissimilatory sulfite reductase (*dsrA*) gene was analyzed as a marker for pre- and post-disturbance SRB (Wagner et al., 2005) potential and the expression of *dsrA* genes by the Salt Pond microbial mat community (**Figure 6**). There were no significant differences (*p* > 0.05) in *dsrA* gene abundance in day/night DNA during both years [day11: (8.2 ± 2.0) × 10^9^, night11: (6.4 ± 1.1) × 10^9^; day12: (1.7 ± 1.4) × 10^9^), night12: (6.2 ± 2.7) × 10^9^]. While gene abundance was equal across conditions, RNA/DNA ratios indicated low-level *dsrA* expression (≤7%) under all conditions except in the 2012 lower salinity day samples. Under the 2012 daytime condition, the average RNA/DNA ratio increased to 81% representing a significant increase (, p < 0.001) in expression. When examining day/night differences in 2012, RNA/DNA ratios indicate that *dsrA* expression was greatest during the day and suppressed at night.

#### Sulfide Oxidation

In this study, we measured the abundance of the *soxB* gene to determine the microbial community sulfide oxidizing potential and activity by the Salt Pond community during day and night under high and low salinity. There were no significant differences in *soxB* gene abundance extracted from day [2011: (5.7 ± 0.7) × 10^8^; 2012: (4.0 ± 1.6) × 10^8^] and night [(2011: 3.1 ± 2.2) × 10^8^; 2012: (1.8 ± 1.1) × 10^8^] samples across years, suggesting the potential for sulfide oxidation exists under both salinity conditions (**Figure 6**). However, no *soxB* genes were detected in RNA isolated from the same samples (other biogeochemical genes were amplified from these samples).

## Discussion

### Microbial Mat Compositional Sensitivity to Disturbance

We used a coastal hypersaline microbial mat ecosystem to examine the sensitivity of a semi-closed community to a pulse disturbance, and to examine the extent to which overall community shifts altered biogeochemical potentials. Community sensitivity was examined through comparative 16S rDNA and rRNA analysis to determine shifts in archaeal and bacterial abundance and metabolic potential. Studies have previously used 16S rRNA:rDNA ratios to infer metabolic activity; however, a number of caveats could potentially confound data interpretation (Blazewicz et al., 2013; Denef et al., 2016). Given the considerations outlined in the aforementioned studies, we used rRNA:rDNA ratios to conservatively estimate the metabolic potential (PSP) of the community. Domain-level sensitivity was first observed, with Bacteria becoming more abundant than Archaea following the post-disturbance reduction in salinity. Differences in beta diversity observed across conditions suggest significant changes in community structure, which further supports studies that have identified salinity as a main determining factor in controlling microbial diversity (Benlloch et al., 2002; Rietz and Haynes, 2003; Tripathi et al., 2005; Lozupone and Knight, 2007; Canfora et al., 2014; Webster et al., 2015; Aanderud et al., 2016). In addition, uniform clustering of rDNA and rRNA distances was observed for all conditions except for the bacterial community at high salinity. These data suggest that the archaeal community may contain habitat generalists with a wide range of osmotic tolerance while the bacterial community consists of more habitat specialists that are selected under different environmental conditions. These results support a previous study that observed selection of bacterial specialists within isolated ecosystems that have adapted to salinity extremes (Logares et al., 2013). OTU-level analysis across conditions was used to further resolve changes in community structure and showed dynamic shifts in rare/abundant OTUs throughout both archaeal and bacterial communities. In addition to increased abundance of large numbers of previously rare OTUs, approximately 46% and 61% of archaeal and bacterial post-disturbance OTUs were detected only under the lower salinity conditions. These data suggest that the microbial mat rare biosphere played a significant role in the post-hurricane adaptation of the ecosystem and further supports other studies suggesting that the rare biosphere provides functional flexibility to ecosystems during periods environmental disturbance (Jones and Lennon, 2010; Lennon and Jones, 2011; Shade et al., 2012; Aanderud et al., 2015; Coveley et al., 2015). Classification of OTUs indicated that rare/abundant shifts occurred broadly across archaeal and bacterial taxonomic ranks, often coinciding with shifts in 16S rRNA:rDNA ratios.

Within the archaeal community, post-disturbance trends were observed wherein richness and abundance significantly decreased for Halobacteria and increased for Thermoplasmata, Crenarchaeota, and Thaumarchaeota following the salinity shift. The decrease in Halobacteria abundance is not surprising as taxa within this class are known to thrive in environments with salinities over 100 g kg^-1^ (Ochsenreiter et al., 2002; Sorensen et al., 2005; Maturrano et al., 2006; Oren, 2008; Youssef et al., 2012; Sorokin et al., 2014; Williams et al., 2014; Najjari et al., 2015). However, while Halobacteria decreased in total abundance, some OTUs shifted from rare to abundant concomitant with increased rRNA:rDNA ratios. Similar to a previous study (Najjari et al., 2015), these shifts suggest salinity-driven succession within the halobacterial community. The increased abundance and PSP of Crenarchaeota in post-disturbance samples suggests that high salinity provided an environmental constraint to OTUs within this taxon. This is consistent with a study that found Crenarchaeota to be dominant in lake sediments of intermediate salinity while Halobacteria became more abundant in lakes with higher salinity (Liu et al., 2016). Thermoplasmata and Thaumarchaeota were observed under pre-disturbance conditions but increased in abundance and PSP following the salinity shift, suggesting greater adaptation to lower salinity. Overall, data show that the pulse disturbance generated dynamic shifts in archaeal OTU abundance and rRNA:rDNA ratios, resulting in numerous rare/abundant shifts and an increase in total archaeal PSP. Some taxa within these groups are thought to be involved in methanogenesis (Paul et al., 2012) and ammonia-oxidation (Brochier-Armanet et al., 2008; Pester et al., 2011), suggesting that post-disturbance conditions may increase archaeal biogeochemical cycling potential within the microbial mat.

Dynamic shifts in abundance and rRNA:rDNA ratios were also observed across bacterial phyla, with Bacteroidetes abundance decreasing while Cyanobacteria and Proteobacteria increased following the reduction in salinity. The shift in Bacteroidetes occurred as a result of the abundant to rare transition of three OTUs classified within the Sphingobacteria, suggesting they represent halophilic taxa similar to those observed in other hypersaline systems (Trigui et al., 2011). Cyanobacteria are considered pioneer species in many microbial mats and show a range of salt tolerance, resulting in salinity-driven specialization and distribution (Braithwaite and Whitton, 1987; Dubinin et al., 1992; Franks and Stolz, 2009). A range of salt tolerance was also suggested for Cyanobacteria in the current study with one OTU remaining abundant across conditions and others increasing from rare to abundant following the post-disturbance salinity reduction. This is consistent with another study that also identified a shift in cyanobacterial taxa within a microbial mat following environmental disturbance (Yannarell et al., 2007). Within the Proteobacteria, OTUs classified within the Chromatiales order and as *Sedimenticola* shifted from rare to abundant following ecosystem disturbance, resulting in a significant increase in the abundance of Gammaproteobacteria. Taxa within Chromatiales have been identified in ecosystems ranging from freshwater to hypersaline (Caumette et al., 1988, 1991; Mesbah et al., 2007; Gomes et al., 2010) but appear to be suppressed at the extreme high salinity found in the pre-disturbance conditions of this study. In addition, *Sedimenticola* are potential denitrifiers using sulfide as electron donor (Russ et al., 2014) and have been previously described in ocean sediments, indicating possible tolerance toward lower salinities. These results are consistent with a previous study showing decreased bacterial diversity under high salinity conditions (Benlloch et al., 2002) and suggest that the reduced salinity occurring after Hurricane Irene provided more favorable environmental conditions which resulted in a significant increase in overall bacterial PSP. Post-disturbance diel trends showed that the increase in PSP was significantly higher during daytime compared to nighttime. Primary productivity has been shown to be suppressed under high salinity, resulting in a breakdown of typical microbial mat redox gradients (Pinckney et al., 1995). The differences in diel PSP observed here is likely explained by the re-establishment of redox gradients and biogeochemical cycles within the mat ecosystem following the reduction in salinity.

### Microbial Mat Functional Sensitivity to Disturbance

Microbial mats are often considered natural bioreactors due to the close microbial metabolic coupling occurring within a millimeter scale and resulting in enhanced biogeochemical cycling (Visscher and Stolz, 2005). For example, the microbial mat investigated in this study grows in an oligotrophic ecosystem requiring community production and recycling of nitrogen and sulfur compounds. The shift in salinity experienced by the mat ecosystem investigated in this study following Hurricane Irene resulted in dynamic changes in the abundance and PSP of archaeal and bacterial taxa. Many of these taxa likely play a role in biogeochemical processes; therefore, we next examined how the post-disturbance community shifts affected expression of nitrogen and sulfur cycling genes. Quantitative PCR was used to determine the abundance of genes and transcripts as a measure of genetic potential and expression, respectively. Other studies have used a similar approach to identify nitrogen and sulfur cycling potential and expression and have observed a correlation between gene expression and process rates (Church et al., 2005; Philippot and Hallin, 2005; Bernhard et al., 2010; Akob et al., 2012; Turk-Kubo et al., 2012; Headd and Engel, 2013; Bowen et al., 2014). However, it should be noted that potential post-transcriptional modifications and temporal decoupling (Chen et al., 1998; Kim et al., 1999; Nogales et al., 2002; van de Leemput et al., 2011) may confound the link between gene expression and process rates and is therefore used in this study as a comparative measure to assess how the pulse disturbance influenced the biogeochemical potential within the microbial mat ecosystem.

### Nitrogen Cycling

To assess how the microbial mat potential for nitrogen fixation shifted across conditions, *nifH* genes and transcripts were examined and RNA:DNA ratios compared. While *nifH* genes were observed under all conditions, expression was repressed under pre-disturbance conditions and significantly increased following the salinity reduction. These results are consistent with a study observing suppression of N_2_ fixation in microbial mats at salinities over 90 g kg^-1^ (Paerl et al., 1993; Pinckney et al., 1995, 2011). In addition, studies of N_2_ fixation have also observed complex diel regulation of *nifH* expression within different ecosystems (Pinckney et al., 1995; Colón-López et al., 1997; Church et al., 2005; Steunou et al., 2008; Severin and Stal, 2010; Woebken et al., 2015). Within the microbial mat community, *nifH* expression was observed only during daytime, suggesting that N_2_ fixation may be coupled to phototrophy through direct (i.e., heterocystous Cyanobacteria) or indirect (i.e., photosynthate utilization by noncyanobacterial diazotrophs) pathways. While the contribution of individual taxa to *nifH* expression was not established in this study, many OTUs within Classes/Orders known to contain diazotrophic members, such as Methanobacteria, Rhizobiales, and Desulfobacterales, demonstrated increased abundance and PSP in daytime samples following the post-disturbance reduction in salinity.

Nitrification was examined through comparative analysis of *amoA* genes and transcripts. Within the microbial mat, no bacterial *amoA* genes were detected; however, amplification of archaeal *amoA* genes across conditions suggests a dominant role of the archaeal community in nitrification within this ecosystem. While *amoA* transcripts were detected in both day and night samples during the high salinity conditions, the transcript abundance increased following the salinity reduction, suggesting increased expression and higher nitrification potential. As discussed earlier, while taxa within Thaumarchaeota showed high PSP at day and night under both salinities, OTU abundance, richness, and PSP shifted under the lower salinity condition and may have played a role in the increased *amoA* expression. Diel trends showed that *amoA* expression was always higher in daytime samples. Given that AOA require molecular oxygen and show possible pH sensitivity (Beman et al., 2011; Gubry-Rangin et al., 2015), the observed differences in diel expression may result from partial physiological constraints as the photosynthetically driven mat shifts from daytime oxic-alkaline conditions to nighttime anoxic–acidic conditions (Revsbech et al., 1983). For instance, one Thaumarchaeota OTU further classified within the Nitrososphaeria class had increased activity during day versus night. Members of this class have been shown to have a preference to alkaline conditions (Gubry-Rangin et al., 2015), suggesting shifts in pH may regulate the activity of this OTU. While archaeal *amoA* genes have been identified in a range of ecosystems (Francis et al., 2005; Stahl and de la Torre, 2012; Yang et al., 2013), the expression observed under the high salinity condition in this study expands the known range of salinity tolerance.

The process of denitrification, reduction of nitrate or nitrite to nitrous oxide or dinitrogen, is the major mechanism by which fixed nitrogen returns to the atmosphere from soil and water.

While both *nirK and nirS* nitrite reductase genes have been identified in microbial mats across a range of salinities (Desnues et al., 2007; Fan et al., 2015), only *nirS* was identified in the mat ecosystem examined in this study. This supports other studies suggesting possible environmentally driven differences in the ecological distribution of *nirK/nirS* genes (Oakley et al., 2007; Smith and Ogram, 2008; Jones and Hallin, 2010). Across both years, the abundance of *nirS* and *nosZ* (nitrous oxide reductase) genes remained similar, suggesting the overall denitrification potential of the community was maintained in both pre- and post-disturbance conditions. Similar to another study that observed decreased gene abundance along the denitrification pathway (Bru et al., 2011), we also observed greater abundance of *nirS* compared to *nosZ*. This difference in abundance is likely linked to the sequential reduction in energy gained by each step (Koike and Hattori, 1975). While gene abundance remained similar across conditions, increased RNA:DNA ratios observed in post-disturbance samples suggest increased gene expression and potential for denitrification with reduced salinity. While rates of denitrification are regulated by many variables (e.g., oxygen concentration, pH, temperature, and salinity), the greatest influence is often considered to be carbon and nitrogen availability (Hallin et al., 2009; Jones and Hallin, 2010; Babbin and Ward, 2013). Extreme salinities have been shown to reduce microbial mat photosynthetic carbon fixation and nitrogen fixation; therefore, the post-disturbance reduction in salinity likely increased carbon and nitrogen availability and enhanced denitrification. The low nighttime expression of these genes suggests that denitrification might be coupled to anoxygenic photosynthetic bacteria as has been observed in lake sediments (Shen and Hirayama, 1991) or that the routine diel changes in pH, oxygen, and carbon availability provide an environmental constraint that limits denitrification to daytime. Post-disturbance community profiles showed that OTUs classified as Halobacteriaceae, Rhodospirllales, and Rhodobacterales significantly increased PSP following the reduction in salinity. Denitrification has been shown by some taxa within these groups (Tomlinson et al., 1986; Yoshimatsu et al., 2000; Hattori et al., 2016), suggesting a possible increasing contribution of these taxa to denitrification within the microbial mat ecosystem.

### Sulfur Cycling

Microbial mats typically have high rates of primary productivity resulting in the production and excretion of photosynthates into surrounding sediments (Bateson and Ward, 1988). Within marine hypersaline systems, these organic compounds are mineralized, in large part, by microbes using anaerobic respiration coupled to sulfate reduction (Baumgartner et al., 2006; Brocke et al., 2015). During both years, no difference in *dsrA* gene abundance was observed, suggesting that the community contains similar sulfate reduction potential across conditions. While genetic potential remained similar, significant increases in RNA:DNA ratios were observed under the lower salinity daytime condition. This result indicates that the high salinity conditions constrained potential community sulfate reduction while lower salinity conditions enhanced potential reduction. Others have also observed decreased sulfate reduction at salt saturation (Oren, 1999; Kulp et al., 2007; Gu et al., 2012). While the exact mechanism of suppression is unclear, it has been hypothesized that sulfate reduction may not supply enough energy for osmoadaptation (Oren, 1999, 2011) or that increased borate concentration occurring at salt saturation may act as a specific inhibitor of this metabolism (Kulp et al., 2007). In addition, 2012 diel trends indicate that *dsrA* expression was greatest during the day and suppressed at night. While sulfate reduction is considered to be a strict anaerobic process, oxygen tolerance has been observed (Cypionka, 1995) and provides a selective advantage by increasing accessibility to carbon derived from photosynthetic processes. This finding is in agreement with other studies that observed high rates of sulfate reduction in the upper layers of microbial mats coupled to carbon fixation by the phototrophic community (Teske et al., 1998). The ability to use sulfur species as an electron acceptor has been identified in a taxonomically diverse group of Archaea and Bacteria. For instance, within the Archaea, all known sulfate-reducing taxa are considered hyperthermophiles and have been classified within the Crenarchaeota, Thermoplasmata, and Methanobacteria (Liu et al., 2012). Within Bacteria, sulfate-reducing taxa are mostly located within the Deltaproteobacteria and Epsilonproteobacteria and have been identified in a wide range of ecosystems, including microbial mats (Muyzer and Stams, 2008). Under the 2011 high salinity condition, while all potential sulfate-reducing groups were active, taxa within the Thermoplasmata had the highest OTU richness and abundance, suggesting these taxa may play a greater role in maintaining the sulfur cycle under extreme salinity (**Figures 1**, **3**, and **5**). Following the post-disturbance reduction in salinity, OTU richness, abundance, and activity increased for all groups concomitant with significant increases in *dsrA* expression. However, OTUs within the Deltaproteobacteria showed the greatest increase in richness and activity during daytime, suggesting a possible shift in the predominant sulfate-reducing group. Overall, it is likely that within this microbial mat ecosystem, salinity influences the major sulfate-reducing taxa with archaeal Thermoplasmata taxa playing a greater role under higher salinities and bacterial Deltaproteobacteria playing a greater role in lower salinity conditions.

Within microbial mat ecosystems, sulfate reduction is closely coupled to the oxidation of reduced sulfur compounds by chemolithotrophic and anoxygenic phototrophic microorganisms utilizing the Sox enzymatic system (Headd and Engel, 2013). Among the Sox system, the *soxB* gene, encoding the periplasmic thiosulfate-oxidizing Sox enzyme, is often used as a marker of microbial community sulfide oxidation potential (Headd and Engel, 2013). In this study, *soxB* genes were detected in all samples while transcripts were undetected. These results suggest that while sulfide oxidation potential exists, the gene is suppressed under all analyzed conditions. The mechanism of this suppression is unclear and is the focus of future studies. However, it is possible that, while sulfide oxidation is thermodynamically favorable for osmoadaptation, increased levels of salinity or other environmental factors may have suppressed the activity of the sulfide oxidizing community. Evidence for a possible salinity-driven suppression of sulfide oxidation was observed in a study examining the sulfur cycle along a salinity gradient from freshwater into the Dead Sea (Häusler et al., 2014). This study observed that increasing salinity selected for different sulfide oxidizing communities with the highest salinity restricting growth to only halotolerant or halophilic taxa. Similarly, while OTUs affiliated with possible sulfide oxidizing groups were detected in Salt Pond samples, their PSP was low across both salinity conditions. Therefore, the sulfide oxidizing community within this ecosystem may be composed of taxa adapted to lower salinity conditions resulting in the suppression of *soxB* expression.

## Conclusion

The microbial mat examined in this study is located within a coastal lagoon ecosystem that experiences routine but wide ranging seasonal variation in environmental conditions. The extensive variability of this system may select for a highly diverse microbial community that provides ecosystem stability following weather-driven disturbances. The ecosystem-level pulse disturbance generated by Hurricane Irene significantly reduced local salinity by 72%, subsequently resulting in Archaea to Bacterial domain shifts. Detailed analysis of this broader trend showed significant shifts among taxa within rare and abundant archaeal and bacterial populations, with less than 1% of taxa remaining abundant. In both archaeal and bacterial communities, the majority of taxa had low PSP across salinities; however, post-disturbance samples showed an increase in the overall microbial mat community PSP, with significant shifts occurring among abundant and rare taxa across and within phyla. While changes in the abundance and PSP of specific taxa can not be linked to individual biogeochemical processes, overall post-disturbance shifts in community structure enhanced expression of genes involved in N and S cycling. Together, these data show the compositional and functional sensitivity of a microbial mat ecosystem to environmental change but also suggest that rare taxa provide a reservoir of genetic diversity that may enhance the functional flexibility of the ecosystem following environmental disturbances. While this is one of the first studies to address how disturbance affects the complex links between microbial diversity and ecosystem processes within hypersaline microbial mat ecosystems, more detailed studies are currently underway and aimed at further defining the ecological principles examined in this study.

## Author Contributions

Microbial mat samples were collected and processed by EP and RN. Laboratory sample processing pipeline was developed by EF and EP. Statistical and computational analysis was performed by EP and RN. Manuscript was reviewed and edited by all authors.

## Conflict of Interest Statement

The authors declare that the research was conducted in the absence of any commercial or financial relationships that could be construed as a potential conflict of interest.

## References

[B1] Aanderud Z. T. Jones S. E. Fierer N. Lennon J. T. (2015). Resuscitation of the rare biosphere contributes to pulses of ecosystem activity. *Front. Microbiol.* 6:24 10.3389/fmicb.2015.00024 PMC4311709 25688238

[B2] Aanderud Z. T. Vert J. C. Lennon J. T. Magnusson T. W. Breakwell D. P. Harker A. R. (2016). Hypersaline lakes harbor more active bacterial communities. *PeerJ* 10.7287/peerj.preprints.1922v1 [Epub ahead of print]. PMC4899617 27375575

[B3] Akob D. M. Lee S. H. Sheth M. Küsel K. Watson D. B. Palumbo A. V. (2012). Gene expression correlates with process rates quantified for sulfate- and Fe(III)-reducing bacteria in U(VI)-contaminated sediments. *Front. Microbiol.* 3:280 10.3389/fmicb.2012.00280 PMC3415069 22908009

[B4] Allen M. A. Goh F. Burns B. P. Neilan B. A. (2009). Bacterial, archaeal and eukaryotic diversity of smooth and pustular microbial mat communities in the hypersaline lagoon of Shark Bay. *Geobiology* 7 82–96. 10.1111/j.1472-4669.2008.00187.x 19200148

[B5] Allison S. D. Martiny J. B. H. (2008). Colloquium paper: resistance, resilience, and redundancy in microbial communities. *Proc. Natl. Acad. Sci. U.S.A.* 105(Suppl. 1) 11512–11519. 10.1073/pnas.0801925105 18695234 PMC2556421

[B6] Auguet J.-C. Barberan A. Casamayor E. O. (2010). Global ecological patterns in uncultured Archaea. *ISME J.* 4 182–190. 10.1038/ismej.2009.109 19847207

[B7] Babbin A. R. Ward B. B. (2013). Controls on nitrogen loss processes in Chesapeake Bay sediments. *Environ. Sci. Technol.* 47 4189–4196. 10.1021/es304842r 23469958

[B8] Bateson M. M. Ward D. M. (1988). Photoexcretion and fate of glycolate in a hot spring cyanobacterial mat. *Appl. Environ. Microbiol.* 54 1738–1743. 16347684 10.1128/aem.54.7.1738-1743.1988 PMC202738

[B9] Baumgartner L. K. Reid R. P. Dupraz C. Decho A. W. Buckley D. H. Spear J. R. (2006). Sulfate reducing bacteria in microbial mats: changing paradigms, new discoveries. *Sediment. Geol.* 185 131–145. 10.1016/j.sedgeo.2005.12.008

[B10] Beman J. M. Chow C.-E. King A. L. Feng Y. Fuhrman J. A. Andersson A. (2011). Global declines in oceanic nitrification rates as a consequence of ocean acidification. *Proc. Natl. Acad. Sci. U.S.A.* 108 208–213. 10.1073/pnas.1011053108 21173255 PMC3017153

[B11] Benlloch S. Lopez-Lopez A. Casamayor E. O. Ovreas L. Goddard V. Daae F. L. (2002). Prokaryotic genetic diversity throughout the salinity gradient of a coastal solar saltern. *Environ. Microbiol.* 4 349–360. 10.1046/j.1462-2920.2002.00306.x 12071980

[B12] Bernhard A. E. Landry Z. C. Blevins A. de la Torre J. R. Giblin A. E. Stahl D. A. (2010). Abundance of ammonia-oxidizing archaea and bacteria along an estuarine salinity gradient in relation to potential nitrification rates. *Appl. Environ. Microbiol.* 76 1285–1289. 10.1128/AEM.02018-09 20038706 PMC2820943

[B13] Blazewicz S. J. Barnard R. L. Daly R. A. Firestone M. K. (2013). Evaluating rRNA as an indicator of microbial activity in environmental communities: limitations and uses. *ISME J.* 7 2061–2068. 10.1038/ismej.2013.102 23823491 PMC3806256

[B14] Boujelben I. Gomariz M. Martínez-García M. Santos F. Peña A. López C. (2012). Spatial and seasonal prokaryotic community dynamics in ponds of increasing salinity of Sfax solar saltern in Tunisia. *Antonie Van Leeuwenhoek* 101 845–857. 10.1007/s10482-012-9701-7 22287033

[B15] Bowen J. L. Babbin A. R. Kearns P. J. Ward B. B. (2014). Connecting the dots: linking nitrogen cycle gene expression to nitrogen fluxes in marine sediment mesocosms. *Front. Microbiol.* 5:429 10.3389/fmicb.2014.00429 PMC4139956 25191309

[B16] Braithwaite C. J. R. Whitton B. A. (1987). Gypsum and halite associated with the cyanobacterium Entophysalis. *Geomicrobiol. J.* 5 43–55. 10.1080/01490458709385956

[B17] Brochier-Armanet C. Boussau B. Gribaldo S. Forterre P. (2008). Mesophilic Crenarchaeota: proposal for a third archaeal phylum, the Thaumarchaeota. *Nat. Rev. Microbiol.* 6 245–252. 10.1038/nrmicro1852 18274537

[B18] Brocke H. J. Wenzhoefer F. de Beer D. Mueller B. van Duyl F. C. Nugues M. M. (2015). High dissolved organic carbon release by benthic cyanobacterial mats in a Caribbean reef ecosystem. *Sci. Rep.* 5:8852 10.1038/srep08852 PMC4649756 25747523

[B19] Bru D. Ramette A. Saby N. P. A. Dequiedt S. Ranjard L. Jolivet C. (2011). Determinants of the distribution of nitrogen-cycling microbial communities at the landscape scale. *ISME J.* 5 532–542. 10.1038/ismej.2010.130 20703315 PMC3105713

[B20] Canfora L. Bacci G. Pinzari F. Lo Papa G. Dazzi C. Benedetti A. (2014). Salinity and bacterial diversity: to what extent does the concentration of salt affect the bacterial community in a saline soil? ed. A. M. Ibekwe. *PLoS ONE* 9:e106662 10.1371/journal.pone.0106662 PMC4154724 25188357

[B21] Caumette P. Baulaigue R. Matheron R. (1988). Characterization of *Chromatium salexigens* sp. nov., a Halophilic Chromatiaceae Isolated from Mediterranean Salinas. *Syst. Appl. Microbiol.* 10 284–292. 10.1016/S0723-2020(88)80014-6

[B22] Caumette P. Baulaigue R. Matheron R. (1991). *Thiocapsa halophila* sp. nov., a new halophilic phototrophic purple sulfur bacterium. *Arch. Microbiol.* 155 170–176. 10.1007/BF00248613

[B23] Chen Y. B. Dominic B. Mellon M. T. Zehr J. P. (1998). Circadian rhythm of nitrogenase gene expression in the diazotrophic filamentous nonheterocystous cyanobacterium *Trichodesmium* sp. strain IMS 101. *J. Bacteriol.* 180 3598–3605. 9658003 10.1128/jb.180.14.3598-3605.1998 PMC107328

[B24] Church M. J. Short C. M. Jenkins B. D. Karl D. M. Zehr J. P. (2005). Temporal patterns of Nitrogenase Gene (nifH) expression in the Oligotrophic North Pacific Ocean. *Appl. Environ. Microbiol.* 71 5362–5370. 10.1128/AEM.71.9.5362-5370.2005 16151126 PMC1214674

[B25] Colón-López M. S. Sherman D. M. Sherman L. A. (1997). Transcriptional and translational regulation of nitrogenase in light-dark- and continuous-light-grown cultures of the unicellular cyanobacterium *Cyanothece* sp. strain ATCC 51142. *J. Bacteriol.* 179 4319–4327. 9209050 10.1128/jb.179.13.4319-4327.1997 PMC179256

[B26] Coveley S. Elshahed M. S. Youssef N. H. (2015). Response of the rare biosphere to environmental stressors in a highly diverse ecosystem (Zodletone spring. OK, USA). *PeerJ* 3:e1182 10.7717/peerj.1182 PMC4548494 26312178

[B27] Cypionka H. (1995). “Solute transport and cell energetics,” in *Sulfate-Reducing Bacteria* ed. Barton L. L. (Boston, MA: Springer) 151–184.

[B28] Denef V. J. Fujimoto M. Berry M. A. Schmidt M. L. (2016). Seasonal succession leads to habitat-dependent differentiation in ribosomal RNA:DNA ratios among freshwater lake bacteria. *Front. Microbiol.* 7:606 10.3389/fmicb.2016.00606 PMC4850342 27199936

[B29] Desnues C. Michotey V. D. Wieland A. Zhizang C. Fourçans A. Duran R. (2007). Seasonal and diel distributions of denitrifying and bacterial communities in a hypersaline microbial mat (Camargue, France). *Water Res.* 41 3407–3419. 10.1016/j.watres.2007.04.018 17590406

[B30] Dillon J. G. Carlin M. Gutierrez A. Nguyen V. McLain N. (2013). Patterns of microbial diversity along a salinity gradient in the Guerrero Negro solar saltern, Baja CA Sur, Mexico. *Front. Microbiol.* 4:399 10.3389/fmicb.2013.00399 PMC3868825 24391633

[B31] Dubinin A. V. Gerasimenko L. M. Gusev M. V. (1992). Physiological features of a strain of Microcoleus chthonoplastes from a hypersaline reservoir. *Mikrobiol.* 61 47–51.

[B32] Edwards U. Rogall T. Blöcker H. Emde M. Böttger E. C. (1989). Isolation and direct complete nucleotide determination of entire genes. Characterization of a gene coding for 16S ribosomal RNA. *Nucleic Acids Res.* 17 7843–7853. 10.1093/nar/17.19.7843 2798131 PMC334891

[B33] Falkowski P. G. Fenchel T. Delong E. F. (2008). The microbial engines that drive Earth’s biogeochemical cycles. *Science* 320 1034–1039. 10.1126/science.1153213 18497287

[B34] Fan H. Bolhuis H. Stal L. J. (2015). Drivers of the dynamics of diazotrophs and denitrifiers in North Sea bottom waters and sediments. *Front. Microbiol.* 6:738 10.3389/fmicb.2015.00738 PMC4508842 26257718

[B35] Francis C. A. Roberts K. J. Beman J. M. Santoro A. E. Oakley B. B. (2005). Ubiquity and diversity of ammonia-oxidizing archaea in water columns and sediments of the ocean. *Proc. Natl. Acad. Sci. U.S.A.* 102 14683–14688. 10.1073/pnas.0506625102 16186488 PMC1253578

[B36] Franks J. Stolz J. F. (2009). Flat laminated microbial mat communities. *Earth Sci. Rev.* 96 163–172. 10.1016/j.earscirev.2008.10.004

[B37] Fraterrigo J. M. Rusak J. A. (2008). Disturbance-driven changes in the variability of ecological patterns and processes. *Ecol. Lett.* 11 756–770. 10.1111/j.1461-0248.2008.01191.x 18422637

[B38] Glasby T. M. Underwood A. J. (1996). Sampling to differentiate between pulse and press perturbations. *Environ. Monit. Assess.* 42 241–252. 10.1007/BF00414371 24193581

[B39] Gomes N. C. M. Cleary D. F. R. Pinto F. N. Egas C. Almeida A. Cunha A. (2010). Taking root: enduring effect of rhizosphere bacterial colonization in mangroves. *PLoS ONE* 5:e14065 10.1371/journal.pone.0014065 PMC2989908 21124923

[B40] Green S. J. Blackford C. Bucki P. Jahnke L. L. Prufert-Bebout L. (2008). A salinity and sulfate manipulation of hypersaline microbial mats reveals stasis in the cyanobacterial community structure. *ISME J.* 2 457–470. 10.1038/ismej.2008.6 18288215

[B41] Gu C. Laverman A. M. Pallud C. E. (2012). Environmental controls on nitrogen and sulfur cycles in surficial aquatic sediments. *Front. Microbiol.* 3:45 10.3389/fmicb.2012.00045 PMC3286789 22375137

[B42] Gubry-Rangin C. Kratsch C. Williams T. A. McHardy A. C. Embley T. M. Prosser J. I. (2015). Coupling of diversification and pH adaptation during the evolution of terrestrial Thaumarchaeota. *Proc. Natl. Acad. Sci. U.S.A.* 112 9370–9375. 10.1073/pnas.1419329112 26170282 PMC4522744

[B43] Hallin S. Jones C. M. Schloter M. Philippot L. (2009). Relationship between N-cycling communities and ecosystem functioning in a 50-year-old fertilization experiment. *ISME J.* 3 597–605. 10.1038/ismej.2008.128 19148144

[B44] Hattori T. Shiba H. Ashiki K. Araki T. Nagashima Y. Yoshimatsu K. (2016). Anaerobic growth of Haloarchaeon *Haloferax volcanii* by denitrification is controlled by the transcription regulator NarO. *J. Bacteriol.* 198 1077–1086. 10.1128/JB.00833-15 26787768 PMC4800875

[B45] Häusler S. Weber M. Siebert C. Holtappels M. Noriega-Ortega B. E. De Beer D. (2014). Sulfate reduction and sulfide oxidation in extremely steep salinity gradients formed by freshwater springs emerging into the Dead Sea. *FEMS Microbiol. Ecol.* 90 956–969. 10.1111/1574-6941.12449 25348393

[B46] Headd B. Engel A. S. (2013). Evidence for niche partitioning revealed by the distribution of sulfur oxidation genes collected from areas of a terrestrial sulfidic spring with differing geochemical conditions. *Appl. Environ. Microbiol.* 79 1171–1182. 10.1128/AEM.02812-12 23220955 PMC3568610

[B47] Jones C. M. Hallin S. (2010). Ecological and evolutionary factors underlying global and local assembly of denitrifier communities. *ISME J.* 4 633–641. 10.1038/ismej.2009.152 20090785

[B48] Jones S. E. Lennon J. T. (2010). Dormancy contributes to the maintenance of microbial diversity. *Proc. Natl. Acad. Sci. U.S.A.* 107 5881–5886. 10.1073/pnas.0912765107 20231463 PMC2851880

[B49] Kim K. Zhang Y. Roberts G. P. (1999). Correlation of activity regulation and substrate recognition of the ADP-ribosyltransferase that regulates nitrogenase activity in *Rhodospirillum rubrum*. *J. Bacteriol.* 181 1698–1702. 10049407 10.1128/jb.181.5.1698-1702.1999 PMC93565

[B50] Koike I. Hattori A. (1975). Energy yield of denitrification: an estimate from growth yield in continuous cultures of *Pseudomonas* denitrificans under nitrate-, nitrite- and oxide-limited conditions. *J. Gen. Microbiol.* 88 11–19. 10.1099/00221287-88-1-11 1151328

[B51] Kozich J. J. Westcott S. L. Baxter N. T. Highlander S. K. Schloss P. D. (2013). Development of a dual-index sequencing strategy and curation pipeline for analyzing amplicon sequence data on the MiSeq Illumina sequencing platform. *Appl. Environ. Microbiol.* 79 5112–5120. 10.1128/AEM.01043-13 23793624 PMC3753973

[B52] Kulp T. R. Han S. Saltikov C. W. Lanoil B. D. Zargar K. Oremland R. S. (2007). Effects of imposed salinity gradients on dissimilatory arsenate reduction, sulfate reduction, and other microbial processes in sediments from two California soda lakes. *Appl. Environ. Microbiol.* 73 5130–5137. 10.1128/AEM.00771-07 17601810 PMC1950999

[B53] Lennon J. T. Jones S. E. (2011). Microbial seed banks: the ecological and evolutionary implications of dormancy. *Nat. Rev. Microbiol.* 9 119–130. 10.1038/nrmicro2504 21233850

[B54] Ley R. E. Harris J. K. Wilcox J. Spear J. R. Miller S. R. Bebout B. M. (2006). Unexpected diversity and complexity of the Guerrero Negro hypersaline microbial mat. *Appl. Environ. Microbiol.* 72 3685–3695. 10.1128/AEM.72.5.3685-3695.2006 16672518 PMC1472358

[B55] Liu Y. Beer L. L. Whitman W. B. (2012). Sulfur metabolism in archaea reveals novel processes. *Environ. Microbiol.* 14 2632–2644. 10.1111/j.1462-2920.2012.02783.x 22626264

[B56] Liu Y. Priscu J. C. Xiong J. Conrad R. Vick-Majors T. Chu H. (2016). Salinity drives archaeal distribution patterns in high altitude lake sediments on the Tibetan Plateau. *FEMS Microbiol. Ecol.* 92 10.1093/femsec/fiw033 [Epub ahead of print]. 26887660

[B57] Logares R. Lindström E. S. Langenheder S. Logue J. B. Paterson H. Laybourn-Parry J. (2013). Biogeography of bacterial communities exposed to progressive long-term environmental change. *ISME J.* 7 937–948. 10.1038/ismej.2012.168 23254515 PMC3635229

[B58] López-López A. Richter M. Peña A. Tamames J. Rosselló-Móra R. (2013). New insights into the archaeal diversity of a hypersaline microbial mat obtained by a metagenomic approach. *Syst. Appl. Microbiol.* 36 205–214. 10.1016/j.syapm.2012.11.008 23352736

[B59] Lozupone C. A. Knight R. (2007). Global patterns in bacterial diversity. *Proc. Natl. Acad. Sci. U.S.A.* 104 11436–11440. 10.1073/pnas.0611525104 17592124 PMC2040916

[B60] Marshall C. W. Ross D. E. Fichot E. B. Norman R. S. May H. D. (2012). Electrosynthesis of commodity chemicals by an autotrophic microbial community. *Appl. Environ. Microbiol.* 78 8412–8420. 10.1128/AEM.02401-12 23001672 PMC3497389

[B61] Maturrano L. Santos F. Rosselló-Mora R. Antón J. (2006). Microbial diversity in Maras salterns, a hypersaline environment in the Peruvian Andes. *Appl. Environ. Microbiol.* 72 3887–3895. 10.1128/AEM.02214-05 16751493 PMC1489619

[B62] Mesbah N. M. Abou-El-Ela S. H. Wiegel J. (2007). Novel and unexpected prokaryotic diversity in water and sediments of the alkaline, hypersaline lakes of the Wadi An Natrun. *Egypt. Microb. Ecol.* 54 598–617. 10.1007/s00248-006-9193-y 17450395

[B63] Mohit V. Archambault P. Lovejoy C. (2015). Resilience and adjustments of surface sediment bacterial communities in an enclosed shallow coastal lagoon, Magdalen Islands, Gulf of St. Lawrence, Canada. *FEMS Microbiol. Ecol.* 91:fiv038 10.1093/femsec/fiv038 25873467

[B64] Muyzer G. Stams A. J. M. (2008). The ecology and biotechnology of sulphate-reducing bacteria. *Nat. Rev. Microbiol.* 6 441–454. 18461075 10.1038/nrmicro1892

[B65] Najjari A. Elshahed M. S. Cherif A. Youssef N. H. (2015). Patterns and determinants of Halophilic Archaea (Class Halobacteria) diversity in Tunisian Endorheic Salt Lakes and Sebkhet Systems. *Appl. Environ. Microbiol.* 81 4432–4441. 10.1128/AEM.01097-15 25911472 PMC4475886

[B66] Nercessian O. Fouquet Y. Pierre C. Prieur D. Jeanthon C. (2005). Diversity of Bacteria and Archaea associated with a carbonate-rich metalliferous sediment sample from the Rainbow vent field on the Mid-Atlantic Ridge. *Environ. Microbiol.* 7 698–714. 10.1111/j.1462-2920.2005.00744.x 15819852

[B67] Nogales B. Timmis K. N. Nedwell D. B. Osborn A. M. (2002). Detection and diversity of expressed denitrification genes in estuarine sediments after reverse transcription-PCR amplification from mRNA. *Appl. Environ. Microbiol.* 68 5017–5025. 10.1128/AEM.68.10.5017-5025.2002 12324352 PMC126436

[B68] Oakley B. B. Francis C. A. Roberts K. J. Fuchsman C. A. Srinivasan S. Staley J. T. (2007). Analysis of nitrite reductase (nirK and nirS) genes and cultivation reveal depauperate community of denitrifying bacteria in the Black Sea suboxic zone. *Environ. Microbiol.* 9 118–130. 10.1111/j.1462-2920.2006.01121.x 17227417

[B69] Ochsenreiter T. Pfeifer F. Schleper C. (2002). Diversity of Archaea in hypersaline environments characterized by molecular-phylogenetic and cultivation studies. *Extremophiles* 6 267–274. 10.1007/s00792-001-0253-4 12215811

[B70] Oren A. (1999). Bioenergetic aspects of halophilism. *Microbiol. Mol. Biol. Rev.* 63 334–348. 10357854 10.1128/mmbr.63.2.334-348.1999 PMC98969

[B71] Oren A. (2008). Microbial life at high salt concentrations: phylogenetic and metabolic diversity. *Saline Systems* 4:2 10.1186/1746-1448-4-2 PMC2329653 18412960

[B72] Oren A. (2011). Thermodynamic limits to microbial life at high salt concentrations. *Environ. Microbiol.* 13 1908–1923. 10.1111/j.1462-2920.2010.02365.x 21054738

[B73] Paerl H. Joye S. Fitzpatrick M. (1993). Evaluation of nutrient limitation of C02 and N2 fixation in marine microbial mats. *Mar. Ecol. Prog. Ser.* 101 297–306. 10.3354/meps101297

[B74] Paerl H. W. Steppe T. F. Buchan K. C. Potts M. (2003). Hypersaline cyanobacterial mats as indicators of elevated tropical hurricane activity and associated climate change. *Ambio* 32 87–90. 10.1579/0044-7447-32.2.87 12733791

[B75] Paul K. Nonoh J. O. Mikulski L. Brune A. (2012). “Methanoplasmatales,” Thermoplasmatales-related archaea in termite guts and other environments, are the seventh order of methanogens. *Appl. Environ. Microbiol.* 78 8245–8253. 10.1128/AEM.02193-12 23001661 PMC3497382

[B76] Pester M. Bittner N. Deevong P. Wagner M. Loy A. (2010). A “rare biosphere” microorganism contributes to sulfate reduction in a peatland. *ISME J.* 4 1591–1602. 10.1038/ismej.2010.75 20535221 PMC4499578

[B77] Pester M. Schleper C. Wagner M. (2011). The Thaumarchaeota: an emerging view of their phylogeny and ecophysiology. *Curr. Opin. Microbiol.* 14 300–306. 10.1016/j.mib.2011.04.007 21546306 PMC3126993

[B78] Philippot L. Hallin S. (2005). Finding the missing link between diversity and activity using denitrifying bacteria as a model functional community. *Curr. Opin. Microbiol.* 8 234–239. 10.1016/j.mib.2005.04.003 15939345

[B79] Pinckney J. Long R. Paerl H. (2011). Structural and functional responses of microbial mats to reductions in nutrient and salinity stressors in a Bahamian hypersaline lagoon. *Aquat. Microb. Ecol.* 62 289–298. 10.3354/ame01475

[B80] Pinckney J. Paerla H. W. Beboutb B. M. (1995). Salinity control of benthic microbial mat community production in a Bahamian hypersaline lagoon. *J. Exp. Mar. Biol. Ecol.* 187 223–237. 10.1016/0022-0981(94)00185-G

[B81] Pinckney J. L. Paerl H. W. (1997). Anoxygenic photosynthesis and nitrogen fixation by a microbial mat community in a bahamian hypersaline lagoon. *Appl. Environ. Microbiol.* 63 420–426. 16535506 10.1128/aem.63.2.420-426.1997 PMC1389512

[B82] Revsbech N. P. Jmgensen B. B. Blackburn T. H. Cohen Y. (1983). Microelectrode studies of the photosynthesis and O_2_, H_2_S, and pH profiles of a microbial mat1. *Limnol. Oceanogr.* 28 1062–1074. 10.4319/lo.1983.28.6.1062

[B83] Rietz D. Haynes R. (2003). Effects of irrigation-induced salinity and sodicity on soil microbial activity. *Soil Biol. Biochem.* 35 845–854. 10.1016/S0038-0717(03)00125-1

[B84] Russ L. Speth D. R. Jetten M. S. M. Op den Camp H. J. M. Kartal B. (2014). Interactions between anaerobic ammonium and sulfur-oxidizing bacteria in a laboratory scale model system. *Environ. Microbiol.* 16 3487–3498. 10.1111/1462-2920.12487 24750895

[B85] Scheffer M. Carpenter S. Foley J. A. Folke C. Walker B. (2001). Catastrophic shifts in ecosystems. *Nature* 413 591–596. 10.1038/35098000 11595939

[B86] Schloss P. D. Westcott S. L. Ryabin T. Hall J. R. Hartmann M. Hollister E. B. (2009). Introducing mothur: open-source, platform-independent, community-supported software for describing and comparing microbial communities. *Appl. Environ. Microbiol.* 75 7537–7541. 10.1128/AEM.01541-09 19801464 PMC2786419

[B87] Schneider D. Arp G. Reimer A. Reitner J. Daniel R. (2013). Phylogenetic analysis of a microbialite-forming microbial mat from a hypersaline lake of the Kiritimati atoll, Central Pacific. *PLoS ONE* 8:e66662 10.1371/journal.pone.0066662 PMC3677903 23762495

[B88] Segata N. Izard J. Waldron L. Gevers D. Miropolsky L. Garrett W. S. (2011). Metagenomic biomarker discovery and explanation. *Genome Biol.* 12:R60 10.1186/gb-2011-12-6-r60 PMC3218848 21702898

[B89] Severin I. Confurius-Guns V. Stal L. J. (2012). Effect of salinity on nitrogenase activity and composition of the active diazotrophic community in intertidal microbial mats. *Arch. Microbiol.* 194 483–491. 10.1007/s00203-011-0787-5 22228487 PMC3354318

[B90] Severin I. Stal L. J. (2010). NifH expression by five groups of phototrophs compared with nitrogenase activity in coastal microbial mats. *FEMS Microbiol. Ecol.* 73 55–67. 20455943 10.1111/j.1574-6941.2010.00875.x

[B91] Shade A. Peter H. Allison S. D. Baho D. L. Berga M. Bürgmann H. (2012). Fundamentals of microbial community resistance and resilience. *Front. Microbiol.* 3:417 10.3389/fmicb.2012.00417 PMC3525951 23267351

[B92] Shade A. Read J. S. Welkie D. G. Kratz T. K. Wu C. H. McMahon K. D. (2011). Resistance, resilience and recovery: aquatic bacterial dynamics after water column disturbance. *Environ. Microbiol.* 13 2752–2767. 10.1111/j.1462-2920.2011.02546.x 21883795

[B93] Shen J. Hirayama O. (1991). Hydrogen photoproduction and denitrification by photosynthetic bacteria isolated from Lake Nakaumi and its vicinity. *J. Ferment. Bioeng.* 72 338–342. 10.1016/0922-338X(91)90083-S

[B94] Smith J. M. Ogram A. (2008). Genetic and functional variation in denitrifier populations along a short-term restoration chronosequence. *Appl. Environ. Microbiol.* 74 5615–5620. 10.1128/AEM.00349-08 18641159 PMC2547028

[B95] Sogin M. L. Morrison H. G. Huber J. A. Mark Welch D. Huse S. M. Neal P. R. (2006). Microbial diversity in the deep sea and the underexplored “rare biosphere”. *Proc. Natl. Acad. Sci. U.S.A.* 103 12115–12120. 10.1073/pnas.0605127103 16880384 PMC1524930

[B96] Sorensen K. B. Canfield D. E. Oren A. (2004). Salinity Responses of Benthic Microbial Communities in a Solar Saltern (Eilat, Israel). *Appl. Environ. Microbiol.* 70 1608–1616. 10.1128/AEM.70.3.1608-1616.2004 15006785 PMC368310

[B97] Sorensen K. B. Canfield D. E. Teske A. P. Oren A. (2005). Community composition of a hypersaline endoevaporitic microbial mat. *Appl. Environ. Microbiol.* 71 7352–7365. 10.1128/AEM.71.11.7352-7365.2005 16269778 PMC1287706

[B98] Sorokin D. Y. Berben T. Melton E. D. Overmars L. Vavourakis C. D. Muyzer G. (2014). Microbial diversity and biogeochemical cycling in soda lakes. *Extremophiles* 18 791–809. 10.1007/s00792-014-0670-9 25156418 PMC4158274

[B99] Sousa W. P. (1984). The role of disturbance in natural communities. *Annu. Rev. Ecol. Syst.* 15 353–391. 10.1146/annurev.es.15.110184.002033

[B100] Stahl D. A. de la Torre J. R. (2012). Physiology and diversity of ammonia-oxidizing archaea. *Annu. Rev. Microbiol.* 66 83–101. 10.1146/annurev-micro-092611-150128 22994489

[B101] Steunou A.-S. Jensen S. I. Brecht E. Becraft E. D. Bateson M. M. Kilian O. (2008). Regulation of nif gene expression and the energetics of N2 fixation over the diel cycle in a hot spring microbial mat. *ISME J.* 2 364–378. 10.1038/ismej.2007.117 18323780

[B102] Teske A. Ramsing N. B. Habicht K. Fukui M. Küver J. Jørgensen B. B. (1998). Sulfate-reducing bacteria and their activities in cyanobacterial mats of solar lake (Sinai, Egypt). *Appl. Environ. Microbiol.* 64 2943–2951. 9687455 10.1128/aem.64.8.2943-2951.1998 PMC106797

[B103] Tomlinson G. A. Jahnke L. L. Hochstein L. I. (1986). *Halobacterium denitrificans* sp. nov., an extremely halophilic denitrifying bacterium. *Int. J. Syst. Bacteriol.* 36 66–70. 10.1099/00207713-36-1-66 11542071

[B104] Trigui H. Masmoudi S. Brochier-Armanet C. Barani A. Grégori G. Denis M. (2011). Characterization of heterotrophic prokaryote subgroups in the Sfax coastal solar salterns by combining flow cytometry cell sorting and phylogenetic analysis. *Extremophiles* 15 347–358. 10.1007/s00792-011-0364-5 21424516 PMC3084946

[B105] Tripathi S. Kumari S. Chakraborty A. Gupta A. Chakrabarti K. Bandyapadhyay B. K. (2005). Microbial biomass and its activities in salt-affected coastal soils. *Biol. Fertil. Soils* 42 273–277. 10.1007/s00374-005-0037-6

[B106] Turk-Kubo K. A. Achilles K. M. Serros T. R. C. Ochiai M. Montoya J. P. Zehr J. P. (2012). Nitrogenase (nifH) gene expression in diazotrophic cyanobacteria in the Tropical North Atlantic in response to nutrient amendments. *Front. Microbiol.* 3:386 10.3389/fmicb.2012.00386 PMC3487379 23130017

[B107] van de Leemput I. A. Veraart A. J. Dakos V. de Klein J. J. M. Strous M. Scheffer M. (2011). Predicting microbial nitrogen pathways from basic principles. *Environ. Microbiol.* 13 1477–1487. 10.1111/j.1462-2920.2011.02450.x 21429064

[B108] Visscher P. T. Stolz J. F. (2005). Microbial mats as bioreactors: populations, processes, and products. *Palaeogeogr. Palaeoclimatol. Palaeoecol.* 219 87–100. 10.1016/j.palaeo.2004.10.016

[B109] Wagner M. Loy A. Klein M. Lee N. Ramsing N. B. Stahl D. A. (2005). Functional marker genes for identification of sulfate-reducing prokaryotes. *Methods Enzymol.* 397 469–489. 10.1016/S0076-6879(05)97029-8 16260310

[B110] Warnes G. Bolker B. Bonebakker L. Gentleman R. Hubert W. Liaw A. (2012). *gplots: Various R Programming Tools for Plotting Data*. Available at: http://cran.r-project.org/package=gplots

[B111] Webster G. O’Sullivan L. A. Meng Y. Williams A. S. Sass A. M. Watkins A. J. (2015). Archaeal community diversity and abundance changes along a natural salinity gradient in estuarine sediments. *FEMS Microbiol. Ecol.* 91 1–18. 10.1093/femsec/fiu025 PMC4399439 25764553

[B112] Whitehead T. (1999). Phylogenetic diversity of methanogenic archaea in swine waste storage pits. *FEMS Microbiol. Lett.* 179 223–226. 10.1111/j.1574-6968.1999.tb08731.x 10518719

[B113] Williams T. J. Allen M. A. DeMaere M. Z. Kyrpides N. C. Tringe S. G. Woyke T. (2014). Microbial ecology of an Antarctic hypersaline lake: genomic assessment of ecophysiology among dominant haloarchaea. *ISME J.* 8 1645–1658. 10.1038/ismej.2014.18 24553470 PMC4817606

[B114] Woebken D. Burow L. C. Behnam F. Mayali X. Schintlmeister A. Fleming E. D. (2015). Revisiting N2 fixation in Guerrero Negro intertidal microbial mats with a functional single-cell approach. *ISME J.* 9 485–496. 10.1038/ismej.2014.144 25303712 PMC4303640

[B115] Yang J. Jiang H. Dong H. Wang H. Wu G. Hou W. (2013). amoA-encoding archaea and thaumarchaeol in the lakes on the northeastern Qinghai-Tibetan Plateau, China. *Front. Microbiol.* 4:329 10.3389/fmicb.2013.00329 PMC3824093 24273535

[B116] Yannarell A. C. Steppe T. F. Paerl H. W. (2007). Disturbance and recovery of microbial community structure and function following Hurricane Frances. *Environ. Microbiol.* 9 576–583. 10.1111/j.1462-2920.2006.01173.x 17298358

[B117] Yoshimatsu K. Sakurai T. Fujiwara T. (2000). Purification and characterization of dissimilatory nitrate reductase from a denitrifying halophilic archaeon, *Haloarcula marismortui*. *FEBS Lett.* 470 216–220. 10.1016/S0014-5793(00)01321-1 10734237

[B118] Youssef N. H. Ashlock-Savage K. N. Elshahed M. S. (2012). Phylogenetic diversities and community structure of members of the extremely halophilic archaea (Order Halobacteriales) in multiple saline sediment habitats. *Appl. Environ. Microbiol.* 78 1332–1344. 10.1128/AEM.07420-11 22179255 PMC3294467

