# Supplementary material for: Microbial Mat Compositional and Functional Sensitivity to Environmental Disturbance
Source: Front Microbiol. 2016 Oct 17;7:1632. doi: 10.3389/fmicb.2016.01632 (PMC5066559; doi:10.3389/fmicb.2016.01632)
Supplement: Supplementary file 1 [file Table_1.PDF]

Supplemental Table S1. MID sequences added to the U529 reverse primer.

ID	MID Sequence	Library 1 2012 rDNA	Library 2 2011 rDNA	Library 3 2012 rRNA	Library 4 2011 rRNA
**MID1**	ACG AGT GCG T	Bac/Arc 10 am 1	Bac/Arc 10 am 1	Bac/Arc 10 am 1	Bac/Arc 10 am 1
**MID2**	ACG CTC GAC A	Bac/Arc 10 am 2	Bac/Arc 10 am 2	Bac/Arc 10 am 2	Bac/Arc 10 am 2
**MID3**	AGA CGC ACT C	Bac/Arc 10 am 3	Bac/Arc 10 am 3	Bac/Arc 10 am 3	Bac/Arc 10 am 3
**MID4**	AGC ACT GTA G	Bac/Arc 5 pm 1	Bac/Arc 5 pm 1	Bac/Arc 5 pm 1	Bac/Arc 5 pm 1
**MID5**	ATC AGA CAC G	Bac/Arc 5 pm 2	Bac/Arc 5 pm 2	Bac/Arc 5 pm 2	Bac/Arc 5 pm 2
**MID6**	ATA TCG CGA G	Bac/Arc 5 pm 3	Bac/Arc 5 pm 3	Bac/Arc 5 pm 3	Bac/Arc 5 pm 3
**MID7**	CGT GTC TCT A	Bac/Arc 10 pm 1	Bac/Arc 10 pm 1	Bac/Arc 10 pm 1	Bac/Arc 10 pm 1
**MID8**	CTC GCG TGT C	Bac/Arc 10 pm 2	Bac/Arc 10 pm 2	Bac/Arc 10 pm 2	Bac/Arc 10 pm 2
**MID9**	CAT AGT AGT G	Bac/Arc 10 pm 3	Bac/Arc 10 pm 3	Bac/Arc 10 pm 3	Bac/Arc 10 pm 3
**MID10**	TCT CTA TGC G	Bac/Arc 5 am 1	Bac/Arc 5 am 1	Bac/Arc 5 am 1	Bac/Arc 5 am 1
**MID11**	TGA TAC GTC T	Bac/Arc 5 am 2	Bac/Arc 5 am 2	Bac/Arc 5 am 2	Bac/Arc 5 am 2
**MID12**	TAC TGA GCT A	Bac/Arc 5 am 3	Bac/Arc 5 am 3	Bac/Arc 5 am 3	Bac/Arc 5 am 3
