# Supplementary material for: Microbial Mat Compositional and Functional Sensitivity to Environmental Disturbance
Source: Front Microbiol. 2016 Oct 17;7:1632. doi: 10.3389/fmicb.2016.01632 (PMC5066559; doi:10.3389/fmicb.2016.01632)
Supplement: Supplementary file 2 [file Table_2.PDF]

Supplemental Table S2. Quantitative PCR Primers for genes involved in biogeochemical processes and taxa used in this study:

Target group / process	Primer set	Amplicon size (bp)	Annealing T_a (°C)	Annealing time (min)	Reference
**N_2 fixation**	*nifH*1F, *nifH*6R	341	51	1:00	(Marusina *et al.*, 2001)
**Nitrification**	arch-*amoA*F, arch-*amoA*R	664	53	1:00	(Francis *et al.*, 2005)
	amoA-1F, *amoA*-2R	491	60	1:30	(Rotthauwe and Witzel, 1997)(Avrahami *et al.*, 2003)
**Anaerobic ammonium oxidation**	*hzo*QPCR1F, *hzo*QPCR1R	224	53	0:45	(Long *et al.*, 2013)
**Denitrification**	*nirS*Cd3aF, *nirS*R3cd	325	60	1:00	(Throbäck *et al.*, 2004)
	*nosZ*1F, *nosZ*1R	179	60	1:00	(Henry *et al.*, 2006)
	nirK876, nirK1040	165	63	0:30	(Henry *et al.*, 2004)
**Sulfate reduction**	*dsr*1F, *dsr*4R	780	54	1:00	(Wagner *et al.*, 1998)
**Sulfide oxidation**	*soxB*693, *soxB*1446	765	55.6	0:30	(Petri *et al.*, 2001)
**Archaea**	arc109F, U529R	420	62	0:45	(Whitehead, 1999)
**Bacteria**	27F, U529R	502	62	0:45	(Edwards *et al.*, 1989)
