# Supplementary material for: Microbial Mat Compositional and Functional Sensitivity to Environmental Disturbance
Source: Front Microbiol. 2016 Oct 17;7:1632. doi: 10.3389/fmicb.2016.01632 (PMC5066559; doi:10.3389/fmicb.2016.01632)
Supplement: Supplementary file 3 [file Table_3.PDF]

**Supplemental Table 3.** Comparison of pre- and post-disturbance mean PSP (rRNA/rDNA ratios) of Archaea classes and Bacteria phyla. One-way analysis of variance (ANOVA) (p=0.05) was used to test the differences of mean PSP among conditions. Student's t-test was used to test total mean PSP between 2011 and 2012.

| **Archaea** | | mean PSP | | | | |
|---|---|---|---|---|---|---|
| **Class** | **OTUs** | **Day 2011** | **Night 2011** | **Day 2012** | **Night 2012** | **significance** |
| **Thaumarchaeota unclassified** | **20** | 0.81 ± 0.27 [a] | 1.30 ± 0.42 [a] | 3.70 ± 0.75 [ab] | 5.20 ± 0.48 [b] | 0.003 |
| **MCG** | **8** | 0.19 ± 0.10 [a] | 1.17 ± 1.16 [a] | 1.06 ± 0.23 [a] | 2.92 ± 0.66 [b] | 0.041 |
| **Crenarchaeota unclassified** | **17** | 0.22 ± 0.1 [a] | 0.71 ± 0.58 [a] | 5.82 ± 2.08 [ab] | 14.52 ± 4.90 [b] | <0.001 |
| **Halobacteria** | **17** | 0.78 ± 0.10 | 0.85 ± 0.13 | 3.00 ± 1.30 | 2.30 ± 0.83 | >0.05 |
| **Methanomicrobia** | **2** | 1.12 ± 0.62 | 0.45 ± 0.39 | 2.12 ± 0.51 | 2.96 ± 1.65 | >0.05 |
| **Thermoplasmata** | **122** | 1.28 ± 0.19 [a] | 0.91 ± 0.18 [a] | 0.95 ± 0.08 [a] | 3.32 ± 0.83 [b] | <0.001 |
| **Euryarchaeota unclassified** | **90** | 2.80 ± 0.60 [a] | 1.76 ± 0.25 [ab] | 0.60 ± 0.12 [b] | 0.52 ± 0.21 [b] | <0.001 |
| **Euryarchaeota uncultured** | **15** | 2.48 ± 0.64 [a] | 1.38 ± 0.34 [ab] | 0.53 ± 0.19 [b] | 0.39 ± 0.18 [b] | <0.001 |
| **total mean PSP** | **291** | 1.66 ± 0.21 [a] | 1.21 ± 0.12 [a] | 1.42 ± 0.16 [a] | 3.01 ± 0.48 [b] | <0.001 |
| **total mean PSP 2011 vs 2012** | | 1.43 ± 0.12 [a] | | 2.22 ± 0.25 [b] | | 0.005 |

| **Bacteria** | | mean PSP | | | | |
|---|---|---|---|---|---|---|
| **Phylum** | **OTUs** | **Day 2011** | **Night 2011** | **Day 2012** | **Night 2012** | **significance** |
| **Acidobacteria** | **6** | 0.21 ± 0.08 [a] | 0.14 ± 0.07 [a] | 2.57 ± 0.90 [b] | 1.04 ± 0.24 [b] | 0.001 |
| **Actinobacteria** | **6** | 0.66 ± 0.19 | 0.58 ± 0.23 | 6.05 ± 3.16 | 0.40 ± 0.19 | > 0.05 |
| **Bacteroidetes** | **62** | 1.35 ± 0.39[ab] | 0.77 ± 0.22 [a] | 3.88 ± 1.37 [b] | 1.30 ± 0.17 [ab] | <0.001 |
| **Chloroflexi** | **3** | 0.07 ± 0.05 | 0.04 ± 0.02 | 0.91 ± 0.38 | 0.62 ± 0.29 | >0.05 |
| **Cyanobacteria** | **32** | 2.44 ± 0.41 [a] | 2.13 ± 0.25 [ab] | 0.85 ± 0.26 [b] | 1.74 ± 0.45 [ab] | 0.01 |
| **Deferribacteres** | **3** | 0.00 ± 0.00 [a] | 0.00 ± 0.00 [a] | 5.53 ± 2.58 [b] | 2.27 ± 0.96 [ab] | 0.01 |
| **Deinococcus-Thermus** | **2** | 1.39 ± 0.57 | 1.09 ± 0.37 | 3.20 ± 2.10 | 0.16 ± 0.07 | >0.05 |
| **Firmicutes** | **1** | 0.00 ± 0.00 [a] | 0.00 ± 0.00 [a] | 0.26 ± 0.16 [a] | 5.27 ± 2.38 [b] | 0.01 |
| **Lentisphaerae** | **5** | 1.13 ± 0.51 | 0.55 ± 0.24 | 2.90 ± 0.97 | 2.09 ± 1.27 | >0.05 |
| **Planctomycetes** | **37** | 0.42 ± 0.08 [a] | 0.41 ± 0.07 [a] | 3.28 ± 0.46 [b] | 1.79 ± 0.31 [c] | <0.001 |
| **Proteobacteria** | **146** | 0.97 ± 0.10 [a] | 1.29 ± 0.16 [a] | 2.76 ± 0.31 [b] | 1.39 ± 0.16 [a] | < 0.001 |
| **Alphaproteobacteria** | **50** | 0.85 ± 0.13 [a] | 1.34 ± 0.35 [ab] | 2.37 ± 0.41 [bc] | 1.02 ± 0.16 [bd] | 0.01 |
| **Deltaproteobacteria** | **75** | 0.98 ± 0.16 [a] | 1.15 ± 0.15 [a] | 3.33 ± 0.52 [b] | 1.65 ± 0.28 [a] | <0.001 |
| **Gammaproteobacteria** | **12** | 0.82 ± 0.22 | 0.93 ± 0.28 | 1.91 ± 0.57 | 1.13 ± 0.40 | >0.05 |
| **Unclassified** | **9** | 1.65 ± 0.51 | 2.61 ± 0.89 | 1.27 ± 0.30 | 1.70 ± 0.54 | >0.05 |
| **Spirochaetes** | **25** | 0.94 ± 0.43 [a] | 0.86 ± 0.31 [a] | 2.22 ± 0.37 [b] | 0.56 ± 0.16 [a] | 0.002 |
| **unclassified** | **49** | 0.14 ± 0.04 [a] | 0.16 ± 0.67 [a] | 4.70 ± 0.74 [b] | 1.28 ± 0.24 [a] | <0.001 |
| **Verrucomicrobia** | **3** | 0.00 ± 0.00 [a] | 0.00 ± 0.00 [a] | 7.53 ± 3.71 [b] | 2.55 ± 0.78 [ab] | 0.02 |
| **total mean PSP** | **380** | 0.90 ± 0.08 [a] | 1.10 ± 0.08 [a] | 3.09 ± 0.26 [b] | 1.33 ± 0.08 [a] | <0.001 |
| **total mean PSP 2011 vs 2012** | | 0.98 ± 0.06 [a] | | 2.13 ± 0.13 [b] | | <0.001 |
