# Supplementary figures and images for: Microbial Mat Compositional and Functional Sensitivity to Environmental Disturbance
Source: Front Microbiol. 2016 Oct 17;7:1632. doi: 10.3389/fmicb.2016.01632 (PMC5066559; doi:10.3389/fmicb.2016.01632)

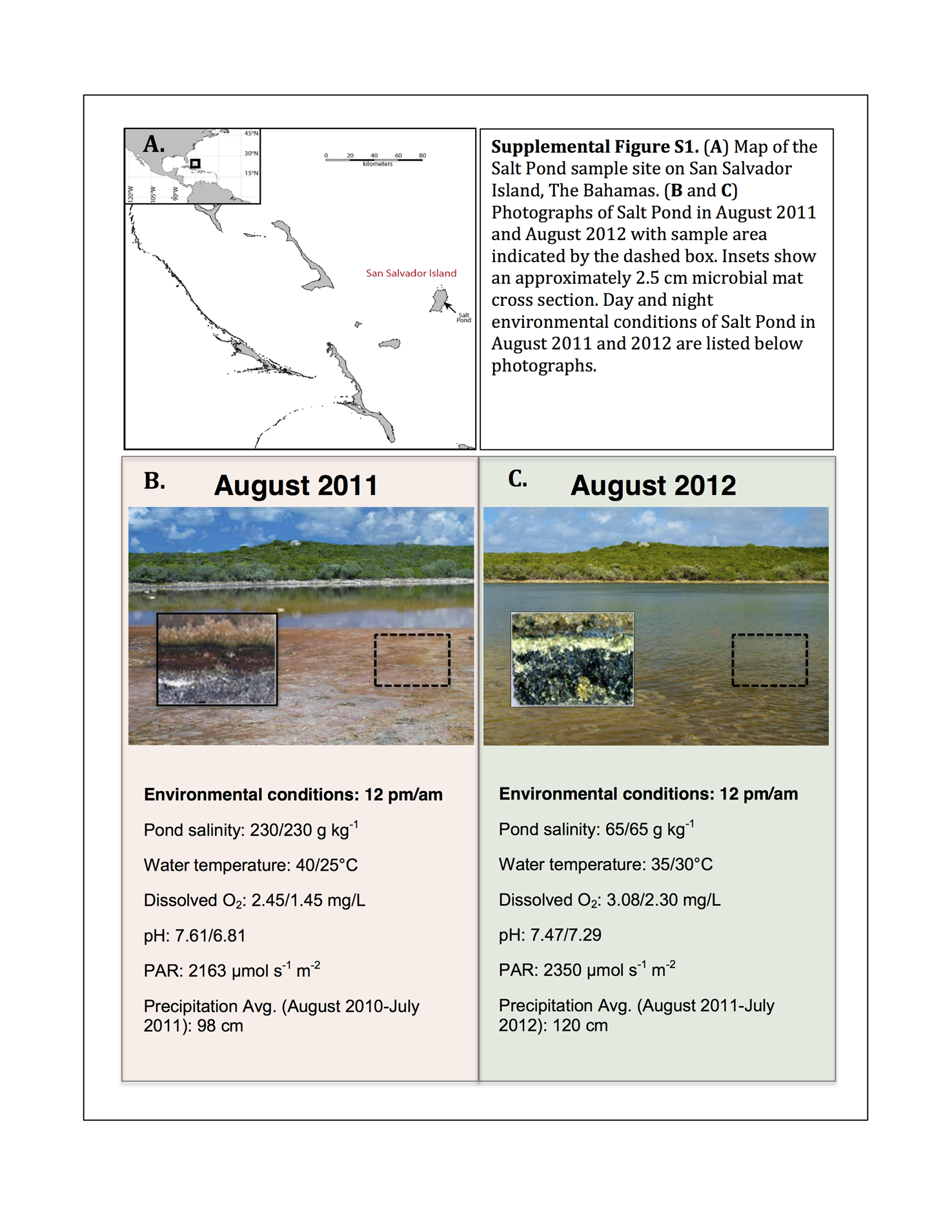

Supplement: Supplementary file 5 [file Image_1.TIF]

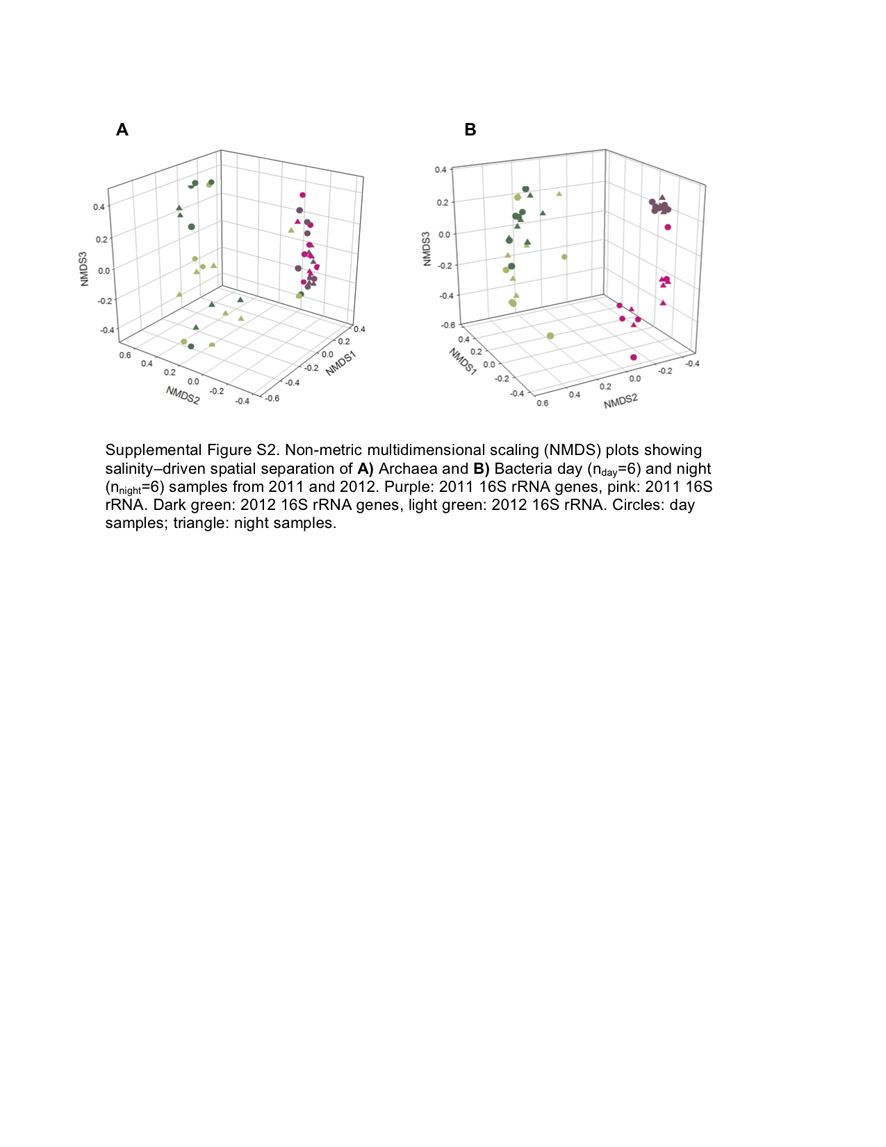

Supplement: Supplementary file 6 [file Image_2.TIFF]
